# Increased human health risks at a legacy mine site: copper and lead bioaccessibility of oxidised tailings

**DOI:** 10.1007/s10653-025-02498-6

**Published:** 2025-07-21

**Authors:** Sean McHale, Heather E. Jamieson, Amy E. Cleaver, Philippa Huntsman

**Affiliations:** 1 Department of Geological Sciences and Geological Engineering, Queen’s University, Kingston, K7L 3 N6 Canada; 2 CanmetMINING, Natural Resources Canada, Ottawa, K1 A 0G1 Canada

**Keywords:** Gastric bioaccessibility, Copper, Lead, Tailings, Mineralogy

## Abstract

With climate change, conditions for dust generation are expected to be more prevalent. A potential dust source of concern are mine wastes due to the likelihood of potentially toxic elements which pose a human health risk if ingested or inhaled. This study analysed sieved mine tailings from a legacy mine in Nova Scotia, Canada. The aim of the research was to analyse total concentrations of Cu and Pb in tailings samples sieved to represent dust; to determine gastric bioaccessibility of Cu and Pb in these samples; and to analyse the impact of mineralogy on Cu and Pb bioaccessibility. Mineralogy was determined with a scanning electron microscope and automated mineralogy software. Tailings were sampled from an uncovered, subaerial, tailings impoundment. Copper bioaccessibility had a strong positive correlation with carbonate and oxide copper hosts. Lead bioaccessibility had a strong positive correlation to one oxidation product, cerussite (lead carbonate). Lead was predominantly hosted in cerussite and had greater bioaccessibility than copper which was predominantly hosted in chalcopyrite. The results highlight the increased human-health risk posed by subaerial tailings at an abandoned mine.

**Supplementary Information:**

The online version contains supplementary material available at 10.1007/s10653-025-02498-6.

## Introduction

Dust has importance as a contaminant transport media due to its potential speed, distance, and aerial extent (Csavina et al., 2012; Morman & Plumlee, 2013). Many sources of dust generation exist: natural sources include deserts, shrublands, desertified lands, dune fields, and playas; anthropogenic examples include landfills, dirt roads, construction, mine tailings (remediated or active), and mining operations (Csavina et al., 2012). Potential dust generation and contaminant transport from both anthropogenic and natural sources is likely to increase as conditions favourable for dust generation are forecasted globally, including high temperatures, increased frequency and duration of droughts, and changes in wind patterns (Csavina et al., 2012; Bush & Lemmen, 2019; IPCC, 2022).

Relative to other dust sources, mining operations and mine tailings are notable because of their potential for particulate emissions with high contaminant concentrations (Csavina et al., 2012). Mine tailings are highly-variable, residue wastes from ore processing, comprised of primary ore and gangue minerals, secondary minerals formed from weathering, chemical precipitates from mineral processing, and chemical precipitates formed after disposal; consequently, tailings management requires site-specific approaches which may be further variable given historic and geographic differences in regulation. Potentially toxic elements, including Sb, As, Cu, Cd, Pb, and Zn, are often associated with mining, whether in primary ore minerals such as metal sulfides, secondary minerals (e.g., metal oxides, carbonates, and sulfates), or in gangue minerals such as carbonates, and iron sulfides (Entwistle et al., 2019; Hudson-Edwards, 2016; Plumlee & Morman, 2011). Exposure to dusts bearing potentially toxic elements can occur by ingestion, inhalation, and skin or eye contact (Plumlee & Morman, 2011).

Studies regarding mining-generated dusts address a variety of aspects, including dispersion, mitigation, health impacts, human-exposure risk, and geochemical characterisation (Drahota et al., 2018; Ettler et al., 2019; Laney & Weissman, 2014; Xu et al., 2019). Mine wastes can be highly heterogeneous: particle-size range may be broad (e.g., < 2.5 µm to boulder size), and mineralisation variable, including both sulfide-rich and -poor material prone to discharging metal(loid)-laden effluents (Entwistle et al., 2019; Lottermoser, 2003; Plumlee & Morman, 2011). Consequently, mine-waste disposal adhering to regulation or environmental stewardship requires a site-specific approach. Indirect disposal methods for tailings, opposed to marine and riverine disposal, includes storage behind cross valley or hillside dams, in tailings storage facilities, as paste backfill into abandoned open pit or underground mines, and dry-stacking on land (Adiansyah et al., 2015; Edraki et al., 2014).

Particles < 60 µm can be transported by suspension (Csavina et al., 2012; Prospero et al., 2002) and are available for inhalation. The human nose filters particles > approximately 10 µm, but the mouth does not (Morman & Plumlee, 2013). Particulate matter between 2.5 and 10 µm can be cleared from the conducting airways (i.e., nose, pharynx, larynx, trachea, bronchi, and bronchioles) to the gastrointestinal tract by the mucociliary elevator (Alpofead et al., 2017); however, particles between 0.5 and 5 µm are generally deposited in the respiratory bronchioles, and between 0.5 and 2.5 µm to the alveoli (Shah et al., 2012). Aside from particle size (as aerodynamic diameter), inflammation of airways, and particle shape factor on deposition in the respiratory system (Carvalho et al., 2011).

Measurements to evaluate the hazard of ingesting or inhaling potentially toxic elements for human health purposes can be made through in vitro testing for bioaccessibility which simulates key gastro, gastro-intestinal, or respiratory processes in humans, or by in vivo testing for bioavailability (Kastury et al., 2017; Ng et al., 2015; Wragg et al., 2018). Lower costs, less time consuming, and lack of animal welfare concerns are advantages of bioaccessibility (in vitro) testing over bioavailability (in vivo) testing (Ng et al., 2015; Ruby et al., 1999). Bioaccessibility is defined as the solubilised concentration of an element from a given material divided by the total concentration of said element from the given material, often expressed as a percentage (Rasmussen et al., 2014). Bioaccessibility testing has been done on a variety of mediums, including art supplies, toys, soils, mine waste, food, house dusts (Cardoso et al., 2015; Palumbo-Roe & Klinck, 2007; Rasmussen et al., 2008).

Bioaccessibility methodology and procedure, as well as mineralogy and particle size, can impact bioaccessibility results. Differences in bioaccessibility methodologies include extraction time, solid-to-fluid ratio, pH, mixing, and constituents and concentrations of extraction fluids (Oomen et al., 2002; Van de Wiele et al., 2007; Juhasz et al., 2009). There has been effort to standardise specific methodologies (Drexler & Brattin, 2007; Wragg et al., 2011), however there is no single standard test for bioaccessibility, or for the reference materials to be used (Koch et al., 2013; Latawiec et al., 2010). Mineralogy and particle size are known impacts on bioavailability and bioaccessibility (Hayes et al., 2012; Li et al., 2021; Meunier et al., 2011).

A variety of methods have been used for mineralogical analysis in studies including bioaccessibility testing, including, X-ray near edge spectroscopy (XANES), X-ray diffraction, Raman spectroscopy, sequential extractions, transmission election microscopy, electron microprobe analysis and scanning electron microscopy with or without automated mineralogy software (Antônio et al., 2021; Cox et al., 2017; Ettler et al., 2014; Gonzalez-Grijalva et al., 2019; Hayes et al., 2012; Helser et al., 2022; Meunier et al., 2010; Molina et al., 2013; Plumlee et al., 2013; Smith et al., 2011). The varied methods have specific advantages and drawbacks, especially when applied to mine wastes which can be highly heterogenous and often contain multiple minerals hosting potentially toxic elements (Hudson-Edwards et al., 2011; Jamieson et al., 2015; Vriens et al., 2020). For example, X-ray diffraction cannot detect amorphous phases, which, in mine wastes, will likely include secondary minerals from weathering, such as iron oxyhydroxides, that tend to adsorb elements such As and Pb, and can facilitate relatively high bioaccessibility and bioavailability (Plumlee & Morman, 2011; Rasmussen et al., 2011; Toujaguez et al., 2013). Consequently, other techniques are often employed alongside X-ray diffraction, such as sequential extractions.

Lead (Pb) bioaccessibility of mine wastes has been widely studied (Bosso & Enzweiler, 2008; Helser et al., 2022; Schaider et al., 2007). Lead sulfide, galena (PbS), the most commercially exploited source mineral for lead (Nayak et al., 2022), is a low-bioaccessible phase: the intra-laboratory study by Drexler and Brattin (2007) reports gastric Pb bioaccessibility for “galena in soil” as 6% ± 2.3; for synthetic galena, Monneron-Gyurits et al. (2020) determined 3.0% Pb gastric and gastro-intestinal bioaccessibility. Plumlee et al. (2013) conclude highly gastric-bioaccessible Pb-carbonate secondary minerals, from weathering of galena, enabled a fatal Pb poisoning outbreak in northern Nigeria, with soil samples from the study having Pb gastric bioaccessibility of 39–66%. Reis et al. (2014) found relatively high gastric Pb bioaccessibility in Pb-hosting Mg–Al-rich carbonate phases compared to Pb-hosting Fe oxyhydroxides; however, decreased Pb bioaccessibility was found in soils with higher carbonate content, as supported by Gonzalez-Grijalva et al. (2019).

Copper (Cu) bioaccessibility in mine wastes has not been as extensively studied as Pb. Driscoll et al. (2011) tested the bioaccessibility of Cu in samples of chalcopyrite (CuFeS_2_), bornite (Cu_5_FeS_4_), malachite (Cu_3_(CO_3_)_2_(OH_2_)), and azurite (Cu_2_(CO_3_)OH_2_). Their aim was to compare Cu solubility in simulated human-body fluids of differing pH: in simulated gastric fluid (pH 1.5 ± 0.05) the carbonate minerals, azurite and malachite, had high Cu bioaccessibility, 72% ± 1 and 66% respectively, the sulfides, bornite and chalcopyrite, had low Cu bioaccessibility, 2% and 0.2% respectively (Driscoll et al., 2011). Furthermore, Cu bioaccessibility was highest for all four minerals in simulated-gastric fluid; their study concluded that metal bioaccessibility observed in their study is specific to extraction-solution pH, mineral form, or both.

The rationale for this study came from the foreseeable conditions favourable for dust generation consequent of climate change, and the need to better understand, using mineralogy, the potential human-health risk posed by an unmanaged contaminant-rich dust source. Furthermore, use of scanning election microscopy with automated mineralogy software in samples analysed for copper mineralogy is a relatively novel approach. The objectives of this research were (i) analyse total concentrations of Cu and Pb in tailings samples sieved to be a proxy for dust, (ii) determine gastric bioaccessibility of Cu and Pb in these samples; and (iii) analyse the impact of mineralogy on Cu and Pb bioaccessibility in these samples.

## Materials and methods

### Site description

Stirling Mine, located in Cape Breton, Nova Scotia, Canada targeted a Zn–Pb–Cu–Au–Ag deposit hosted in pyrite-rich laminated siltstone-chert-dolomite rocks, containing fine-grained pyrite and sphalerite with minor chalcopyrite, galena, and tennantite. Initially the mine operated from 1935 to 1938 under the British Metals Corporation, and 196,000 t of ore with average grades of 9.55% Zn, 2.28% Pb, and 0.94% Cu was mined (Hulshof & Macdonald, 1998). The mine re-opened from 1952 to 1956 under the Mindamar Metals Corporation, who mined 863,000 t of ore, averaging 5.54% Zn, 1.30% Pb, and 0.69% Cu (Hulshof & Macdonald, 1998). Production stopped due to a decrease in ore grades, thereafter the mine was abandoned (Hulshof & Macdonald, 1998; Jacques Whitford Environment Limited, 1996).

In the 1930 s tailings were deposited in Strachans Brook. Later, in the 1950’s, they were deposited into a tailings impoundment, as required by regulation; the impound was 600 m by 200 m, on the east side of the mine, adjacent to and north of Strachans Brook, with the average thickness of tailings of 4 m (Hulshof & Macdonald, 1998; Jacques Whitford Environment Limited, 1996). Hulshof and Macdonald (1998) noted two types of interstratified sediment in the tailings impoundment: orange pyritic sandy silt and blue-grey pyritic silty mud, the orange color being associated with iron oxyhydroxide coatings consequent of oxidation (Hulshof & Macdonald, 1998). Orange tailings were determined to be relatively pyrite-rich (51 ± 6 wt.%) compared to the blue-grey (22 ± 3 wt.%), and tailings of other distinct colors are present in areas of runoff and at depth (Cleaver et al., 2021). The mine site is currently abandoned, however there are all-terrain vehicle tracks across the tailings impoundment (Fig. S1) which may indicate recreational use of the site, which could lead to dust generation and exposure. Aerial erosion of the impoundment was evident in 2017 and 2018 (Cleaver et al., 2021).

### Sample collection and preparation

This study’s eight tailings samples (TS1a, TS1b, TS2, TS3, TS5, TS6, TS9a, TS9b) were collected in 2017 and 2018 by Cleaver et al. (2021) for a study on the geochemistry and mineralogy of windblown dust. A flow chart summarizing sample prep has been made available (Fig. S2). Samples were from six locations on the tailings impoundment, targeting locations downwind where windblown tailings dust accumulation was evident. Most samples represented surface material, though TS1b and TS9b were taken at ~ 5 cm and 10–25 cm depth respectively. Approximately 0.5–5 kg of sample was taken to allow for reasonable material availability for each size fraction (i.e. < 63um) for subsequent geochemical and mineralogic analysis. During sampling, tailings were categorized by appearance as relatively “pyrite-rich” or “pyrite-poor” (Cleaver, 2020): pyrite-rich tailings (*n* = 5: TS1a, TS2, TS3, TS6, TS9a) were brown-orange and fine to very-fine sand, pyrite-poor tailings were blue-grey clay-silt (*n* = 2: TS1b and TS5). Automated mineralogy determined pyrite-rich tailings contained 51.1 ± 6.1 wt.% pyrite, and pyrite-poor tailings 22.0 ± 3.0 wt.% pyrite. Sample TS9b was not used in Cleaver (2020) and consequently was not assigned as either pyrite-poor or pyrite-rich.

Preparation of samples by Cleaver (2020) included nitrogen drying of wet samples (TS1a, TS1b, TS3, TS6, TS9a, TS9b) by circulation of nitrogen gas (99.998–99.999%) through a glove box for preservation of in-situ phases; dried samples were sieved to the < 63-µm size fraction, so as to be a proxy for dust. Sieves were cleaned by gentle brushing, an ultrasonic cleaner, and compressed air. A micro-rotary riffler was then used by Cleaver (2020) to take numerous 1-g subsamples for geochemical analyses of their study. Remaining sieved and riffled sample from Cleaver (2020) was then used in this study. The tailings samples were subsampled at CanmetMINING using a Microscal Ltd spinning riffler with a Syntron Magnetic Feeder to lower sample into the riffler, these were cleaned between samples using paper towel; elemental analysis, bioaccessibility, and mineralogical analysis used subsamples from this riffling process. Riffling was done to reduce heterogeneity of tailings samples and facilitate comparison and interpretation of bioaccessibility results based on mineralogical characterization of the samples.

### Elemental analysis of tailings

At CanmetMINING, Natural Resources Canada, subsamples of the homogenized tailings samples were digested with aqua regia (6 mL hydrochloric acid and 2 mL nitric acid) in a sealed vessel by microwave heating. Digested samples were analysed on an Agilent 5110 SVDV ICP-OES for Cu, Pb, Zn, and As; and on a Thermo-Fisher i-Cap ICP-MS for Cd and Sb concentrations. One instrumental duplicate was analysed during the elemental analysis of the tailings, see the quality assurance and quality control section. Certified reference materials included CRM-SOIL-A-500 (Soil Solution A) and CRM-SOIL-B-500 (Soil Solution B) from HPS (high-purity standards), and TMDA-54.6, a certified liquid reference material of enriched lake water (fortified with trace metals) from Environment and Climate Change Canada (ECCC). SOIL-B and TMDA-54.6 were analysed for Cu; and for Pb, SOIL-A, SOIL-B and TMDA-54.6; a list of certified references is provided in the supplementary information (Table S1).

### Gastric bioaccessibility

Bioaccessibility testing was done at CanmetMINING using a modified 0.07 M hydrochloric (HCl) method as described by Rasmussen et al. (2008): a gastric simulation using 50 mL 0.07 M HCl, with sample loadings of 10 ± 0.5 mg and 25 ± 1.25 mg, at pH 1.5 ± 0.1 for a 2-h incubation period at 37 °C with 100 rpm agitation of experiment vessels for the first hour and none thereafter. This method is similar to the regulatory 0.07 M HCl gastric assays, e.g., ASTM D5517 (American Society for Testing and Materials, 2021), with two exceptions. First, more dilute fluid-to-solid ratios (5000:1 and 2000:1 ml/g respectively) were selected to avoid potential proton exhaustion or saturation of the extraction solution. Second, either 2 M NaOH or 1 M HCl was used to amend pH (to pH 1.5 ± 0.1) one minute after incubation started, to allow for the assessment of mineralogical controls on metal release with minimal pH variability. Recognizing that regulatory 0.07 M HCl protocols do not use NaOH (and prescribe 1 M HCl only, to adjust pH < 1.5), we re-checked pH at the end of the test to ensure that H^+^ ions were not exhausted during incubation. Unlike Henderson et al. (2014), who adjusted pH using NaOH before the sample was added, we delayed pH amendment until after the sample was mixed with the extraction fluid, consistent with the 0.07 M HCl regulatory protocols.

All extractions for a given sample loading were done in triplicate, with a fourth vessel used as a control to monitor temperature and pH at the start and end of the incubation period; if pH in the control deviated from pH 1.5 ± 0.1, drops of 2 M NaOH or 1 M HCl were added by Eppendorf pipette to return pH to 1.5 ± 0.1, this amendment was then applied to the associated triplicate. Both temperature and pH were measured at the end of the test. Extraction solutions were filtered using pre-wetted 0.2 µm Pall Acrodisc filter syringes on Henke-Sass Wolf syringes to obtain ~ 12 mL aliquots. Eighteen aliquots were taken per sample: two sample loadings done in triplicate, with three aliquots taken per triplicate.

Extraction-solution aliquots were tested at CanmetMINING. All Cu concentrations were analysed with a Thermo-Fisher i-Cap ICP-MS. For Pb, all solutions from 10-mg sample loadings apart from for TS1a were analysed with a Thermo-Fisher i-Cap ICP-MS, the remaining aliquots (from 10- and 25-mg loadings of TS1a, and 25-mg loadings of TS2, TS5, TS6, TS9a) were analysed by Agilent ICP-OES 5110 VDV. The highest limit of quantification for Cu on the ICP-MS was 0.02 µg kg^−1^. The highest limit of quantification for Pb on the ICP-MS was 1.8 µg kg^−1^, and 0.16 mg kg^−1^ on the ICP-OES.

For quality control and quality assurance, triplicates of three blanks (50 mL HCl) and one positive control were analysed along with each sample. The blank was comprised of 50 mL 0.07 M HCl, and the positive control of 25 mg of sample spiked with Cd, Cu, Sb, Pb, and Zn. Spike solutions added were copper chloride (for 53.125 mg/L Cu), lead acetate (155.69 mg/L Pb), zinc chloride (500 mg/L Zn), cadmium chloride (1.555 mg/L Cd), and antimony potassium tartrate (3.11 mg/L Sb). Blanks for bioaccessibility testing were analysed by ICP-MS, using an Agilent 8800 QQQ for sample TS1b analyses, and a Thermo-Fisher i-Cap for blanks of the remaining seven samples. Spikes were analysed by ICP-OES on an Agilent ICP-OES 5110. One instrumental duplicate was analysed per sample loading per sample. For certified reference materials analysed for instrumental accuracy see (Table S3, Table S4). Additionally, the certified reference material NIST 2710a was also tested, to evaluate the accuracy of the bioaccessibility procedure outlined above.

The bioaccessible proportion of an element was calculated as in (Rasmussen et al., 2014):

$$\%_{bio} = \frac{{E_{S} }}{{E_{T } }} \times 100$$

where $E_{S}$ is the bioaccessible concentration of an element, that is divided by $E_{T }$, the total elemental concentration of that element in the sample, multiplied by 100 to calculate $\%_{bio}$,the bioaccessibility (%).

### Mineralogy

Thin sections of the tailings samples (*N* = 8) were made at Queen’s University from the tailings subsamples as follows: each sample was vacuum embedded into EpoTek301 epoxy, forming a 1-cm high cylinder, that was then cut, ground, polished, and mounted onto glass sourced from Beta Diamond Products. Slides were analysed on a FEI Quanta 650 FEG (field emission gun) environmental scanning election microscope (ESEM) at Queen’s University. Automated mineralogy software, Mineral Liberation Analysis (MLA), mapped the thin sections mineralogically, collecting back-scatter election (BSE) and energy dispersive X-ray (EDS) data for each particle detected. Identification of solid phases requires creation of a mineral reference library; for this study, phases in the library were informed by Cleaver et al. (2021), the previous study on these samples. Grains were checked by qualitative observation and grain-by-grain SEM–EDS analyses. On finalizing the mineral library, particles labelled as unknowns were below 2.5 wt.% in all samples. Once the thin section is mapped, the software can be used to analyse many factors including modal mineralogy, element deportment (distribution of an element amongst mineral), particle-size distribution, mineral grain-size distribution, mineral liberation, and mineral association. Scanning election microscopy with mineral liberation analysis software (SEM-MLA) is a semi-quantitative technique, requiring the identification of peaks on energy-dispersive X-ray spectra, the technique excels at identifying solid phases with distinct, simple spectra (e.g., sulfides) but is limited in analysis of chemically similar phases and those with highly variable stoichiometry (Lougheed et al., 2020). The technique has inherent uncertainty from being a two-dimensional analysis of (three-dimensional) particles.

Copper and lead deportment of thin sections was calculated with the equation:

$$Y_{\% }^{{h_{x} }} = \frac{{A^{{h_{x} }} \times t^{{h_{x} }} \times \delta^{{h_{x} }} \times C_{Y}^{{h_{x} }} }}{{{\Sigma }_{h = 1}^{n} m_{Y}^{h} }} \times 100$$

where $Y_{\% }^{{h_{x} }}$ is the proportion of Cu or Pb ($Y$) by mass within a given host phase, $h^{x}$. The numerator is the mass fraction of Cu or Pb for a given host phase: $A^{{h_{x} }}$ the surface area of the given host phase, $t^{{h_{x} }}$ is the assumed thickness of the sample mounted (1 µm), $\delta^{{h_{x} }}$ being density of the host phase, $C_{Y}^{{h_{x} }}$ is the weight percent of Cu or Pb in the host phase. The denominator, $\Sigma_{h = 1}^{n} m_{Y}^{h}$, represents the total mass of Cu or Pb in all identified host phases. Concentration of Cu and Pb in host phases as well as number of point measurements by electron microprobe analyses by Cleaver et al. (2021) can be seen in Table 3 of their study.

### Quality assurance and quality control

#### Copper and Lead concentrations in tailings

Recovery of Pb and Cu from analytical laboratory CRMs was within 95–105% of certified values (Table S2). One instrumental duplicate was analysed for the eight tailings samples, this had a 3.9(%) relative percent difference (RPD) for Cu, and 3.5(%) RPD for Pb (Table S3).

#### Bioaccessibility testing

Bioaccessibility of a standard reference material, NIST 2710a, was first tested: coefficients of variation for between-vessel and within-vessel were ≤ 10% for Cd, Cu, Pd, and Zn. Nickel had a between-vessel coefficient of variation < −100% for the 10-mg loading, this was due to the mean blank Ni concentration being larger than the Ni extract from the NIST2710a. Element recovery from analytical laboratory CRMs analysed alongside NIST 2710a extract solutions was within ± 10% of certified values apart from one value for Cu (111% recovered) from the CRM TMDA-51.5 (Table S4). Bioaccessibility results for NIST 2710a are shown in (Table S5), with comparison to other studies and brief method summaries. All studies used in the comparison of NIST 2710a bioaccessibility results have relatively large sample-to-fluid ratios, 10 g/L or above (Boros et al., 2017; Cao et al., 2019; Nelson et al., 2013; Ono et al., 2016; Paltseva et al., 2018; Pelfrêne & Douay, 2018; Xia et al., 2016), this study uses 0.2 and 0.5 g/L. Further variations include incubation time, extraction fluid, pH, and the number of stages of the extraction process (e.g., gastric, gastrointestinal). Higher Cu bioaccessibility results in our study may be consequent of the smaller solid-to-fluid ratio employed and slightly lower pH of simulated gastric fluid than in (Boros et al., 2017); for Pb, two of the six studies used for comparison reported higher Pb bioaccessibility values when testing NIST 2710a, this may have occurred due to lower pH values (1.2 compared to 1.5 of this study). Comparison to other results is hindered by method variability and a lack of data.

The relative percent difference between instrument duplicates of bioaccessibility extract solutions for both Cu and Pb were within ± 5% (Fig. S3). Generally, RPDs within ± 20% for duplicates are acceptable (Guney et al., 2017; Morman et al., 2009). Concentration values for analytical laboratory CRMs analysed with the bioaccessibility extract solutions from sample testing (opposed to NIST 2710a testing) were mostly within a ± 10% range (26 of 30 values), 29 of 30 were within ± 20%, one value, for Cu recovered from Trace Metals in Drinking Water (TMDW) exceeded ± 20% from the certified value, having had a 77% recovery (Table S6).

Metal recovery from positive controls (spikes) is summarized in (Fig. S4). Copper had a mean recovery of 91.2% ± 3.4, a median of 91.5%, and a range of 84.9–95.7%; for Pb positive controls, mean recovery for spikes was 98.8% ± 5.6, and median 98.4%, recovery ranged from 87.3 to 106.9%. The mean value of blanks exceeded the LOQ for Cu (0.2 µg kg^−1^) for six of eight samples; for Pb, mean blank concentrations exceeded the LOQ (1.8 µg kg^−1^) for seven of eight samples (Table S7). Where blank concentrations exceeded the LOQ, bioaccessibility results had the blank value subtracted. Blank concentrations were two orders of magnitude less than Cu and Pb concentrations extracted from tailings samples.

Temperature and pH readings taken during bioaccessibility testing of the tailings (Table 1) meet the ASTM (2021) requirements of an initial pH between 1.0 and 1.5 and a maintained temperature of 37 ± 2 °C. Initial pH values between samples had greater range than final pH values for both the 10- and 25-mg sample loadings: for 10-mg loadings there is a 0.12 range in initial pH values between samples, compared to the 0.03 range in final pH values between samples; for 25-mg loadings the respective ranges are 0.14 and 0.06.

**Table 1:** Initial (i) and final (f) values for temperature and pH during bioaccessibility testing

	Sample							
	TS1a	TS1b	TS2	TS3	TS5	TS6	TS9a	TS9b
pH i 10 mg	1.3	1.27	1.32	1.37	1.29	1.31	1.3	1.39
pH f 10 mg	1.52	1.53	1.53	1.53	1.53	1.5	1.52	1.52
pH i 25 mg	1.25	1.23	1.27	1.35	1.3	1.28	1.28	1.37
pH f 25 mg	1.52	1.52	1.52	1.56	1.53	1.5	1.54	1.53
°C i 10 mg	37 ± 1	37 ± 1	37 ± 1	37 ± 1	37 ± 1	37 ± 1	37 ± 1	37 ± 1
°C f 10 mg	37.0	37.0	37.0	37.0	37.0	37.0	37.0	37.0
°C i 25 mg	37 ± 1	37 ± 1	37 ± 1	37 ± 1	37 ± 1	37 ± 1	37 ± 1	37 ± 1
°C f 25 mg	37.0	36.8	36.9	37.1	36.9	37.0	37.0	36.9

Bioaccessibility results were considered acceptable as the coefficient of variation for values within vessel were < 10% for measurements for Cu and < 5% for Pb, in addition to the coefficient of variation between vessels being ≤ 15% for Cu and < 5% for Pb. Table 2 shows maximum values of Cu and Pb between-vessel and within-vessel coefficients of variation. All data is within criteria; coefficients of variation were lower in 25-mg loadings than respective 10-mg loadings, and lower in within-vessel measurements compared to between-vessel.

**Table 2:** Maximum values for coefficients of variation in bioaccessibility testing triplicates

	Coefficient of variation (%) max. values			
	Copper		Lead	
	Between-vessel	Within-vessel	Between-vessel	Within-vessel
10-mg loading	15.2	9	10.3	3.6
25-mg loading	7	4.3	6.2	2.3

### Statistical analysis

Correlations and normality were computed using the programming language R (R Core Team, 2023). Normality of Cu and Pb concentrations from elemental analysis of the sieved tailings samples, and Cu and Pb concentrations from elemental analysis of aliquots from bioaccessibility testing was determined using the in-built function “shapiro.test()” to perform a Shapiro–Wilk test using a 0.05 significance level. Based on this outcome correlations were calculated, with the in-built function “cor.test()”, using either Spearman’s rank correlation coefficient or the Pearson correlation coefficient. Concentration and deportment data was plotted using the ggplot2 package (Wickham, 2016).

## Results

### Copper and lead concentrations in tailings

In this study Cu concentrations across the eight tailings samples ranged from 1020 to 2090 mg kg^−1^. Lead concentrations ranged from 3550 to 11,100 mg kg^−1^. Average values for Cu and Pb concentrations in the tailings were at least one order of magnitude higher than CCME soil guidelines for both parkland and industrial sites (Table 3). Copper values for the tailings did not show evidence of non-normality, with the Shapiro–Wilk normality test returning a p-value of 0.530; distribution of Pb values departed significantly from normality, the Shapiro–Wilk normality test computed a *p*-value of 0.001. A strong positive correlation between Cu and Pb concentrations was computed using Spearman’s rank correlation, ρ of 0.90 and *p*-value of 0.005. Summary of elemental analysis, as in Table 3, with Sb, As, Cd, and Zn included is available in Table S8.

**Table 3:** Summary of elemental analysis for Cu and Pb in Stirling tailings, with guideline values from the Canadian Council for Ministers of the Environment (CCME)

Element	Avg. this study* Sieved < 63 µm (mg kg^−1^)	Avg. Cleaver et al., 2021* Sieved < 63 µm (mg kg^−1^)	CCME Soil Guideline Parkland (mg kg^−1^)	CCME Soil Guideline Industrial (mg kg^−1^)
Number of samples	*N* = 8	*N* = 7		
Zn	15,611 ± 7,419	13,529 ± 4,626	250	410
**Pb**	**5226 ± 2470**	3881 ± 1102	140	600
**Cu**	**1549 ± 328**	1631 ± 277	63	91
As	222 ± 43	232 ± 59	12	12
Sb	79 ± 15	89 ± 12	20	40
Cd	39 ± 18	38 ± 10	10	22

*Sample sets between this study and Cleaver et al., 2021 differ, this study includes samples TS9a and TS9b and omits a sample, TS4

The highest concentration for both Cu and Pb occurred in sample TS1a, Pb concentrations in TS1a (11,100 mg kg^−1^) are more than double of the next highest sample, TS3 (5370 mg kg^−1^). Sample TS9b, neither classified as pyrite-rich or pyrite-poor, had the lowest Cu and Pb concentrations. Pyrite-poor samples had relatively low concentrations of Cu and Pb: TS5 had the second lowest concentrations of Cu and Pb, 1,180 mg kg^−1^ and 3560 mg kg^−1^, respectively; TS1b had Cu and Pb concentrations of 1540 mg kg^−1^ and 4390 mg kg^−1^, respectively, which were both slightly less than respective medians (Cu 1600 mg kg^−1^ median, Pb 4470 mg kg^−1^ median).

This study uses Stirling tailings samples collected by Cleaver (2020) and Cleaver et al (2021). Relative percent difference for Cu and Pb concentrations determined for the same samples between our study and Cleaver et al. (2021) are shown in Table 4. Copper concentrations differed less than Pb: seven of eight samples had relative percent difference within 10% for Cu, and for Pb, seven of eight samples had relative percent difference within 17%. One sample, TS5, had high relative percent difference for Cu and Pb, 50% and 65% respectively. Copper concentrations were lower in six of eight samples compared to concentrations in Cleaver et al. (2021); Pb concentrations were higher in seven of eight samples.

**Table 4:** Relative percent difference for copper and lead concentrations between Stirling tailings between this study and Cleaver (2020)

Element	RPD of Tailings between this Study and Cleaver (2020)							
	TS1a	TS1b	TS2	TS3	TS5	TS6	TS9a	TS9b
Cu	1.9	− 2.6	− 9.1	1.2	− 49.7	− 0.6	− 4.2	− 7.0
Pb	5.6	1.1	− 10.6	14.6	64.7	10.4	16.2	10.1

### Mineralogy

The average modal mineralogy of samples classed as either pyrite-poor or pyrite-rich differed from respective results in Cleaver et al. (2021) (Table S9); minerals with relatively high specific gravity (e.g., pyrite, sphalerite, and barite) were more prevalent in the sub-samples mineralogically analysed in this study. Sample heterogeneity and the low wt.% values of individual Cu and Pb hosts (< 0.5 wt.% average) likely impacted this difference in modal mineralogy. The use of SEM-MLA was crucial to identifying secondary mineral phases in this study. Chemical formulas for Cu and Pb hosts can be seen in Table 5.

**Table 5:** Mineral hosts of copper and lead in Stirling tailings. Trace values from electron microprobe analysis in Cleaver (2020)

Mineral	Chemical formula	Avg. Cu (wt.%)	Avg. Pb (wt.%)
Aurichalcite	(Zn, Cu)_5_(CO_3_)_2_(OH)_6_	12	2.9
Cerussite	PbCO_3_	0.2	78
Chalcopyrite	CuFeS_2_	34	–
Galena	PbS	–	86
Goethite	Fe^3+^O(OH)	1	7.8
Hydrohetaerolite	Zn_2_Mn^3+^_4_O_8_•(H_2_O)	1.4	3
Pb-Mn phases	Possibly PbMn_3_O_6_(OH)_2_	3.4	28
Smithsonite	ZnCO_3_	0.3	1.3
Sphalerite	ZnS	0.04	–
Tennantite	Cu(Cu_4_X_2_)As_4_S_12_S'X'= Fe^2+^ or Zn	41	–

**Fig. 1:**
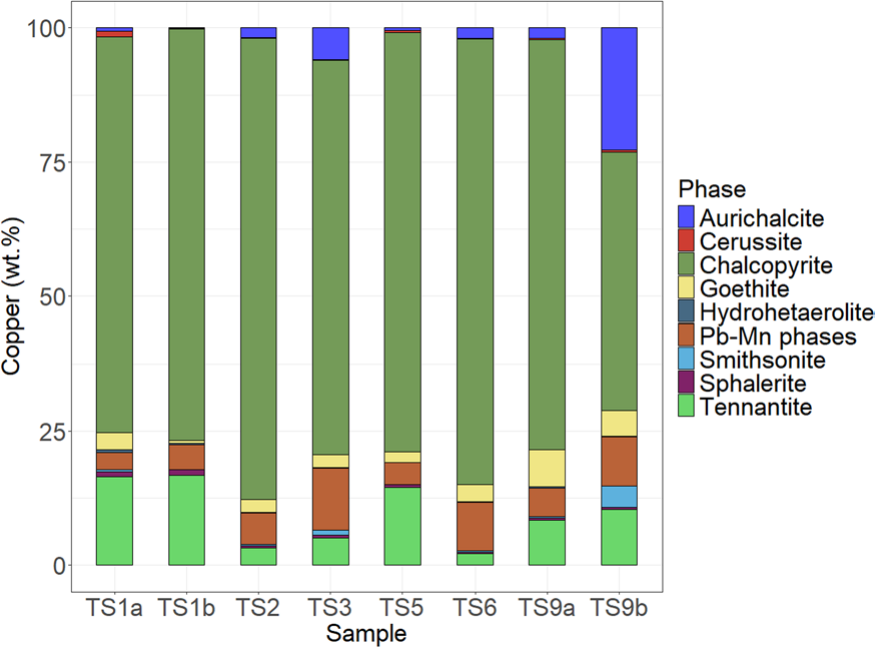
Copper deportment of Stirling tailings samples (N = 8)

**Fig. 2:**
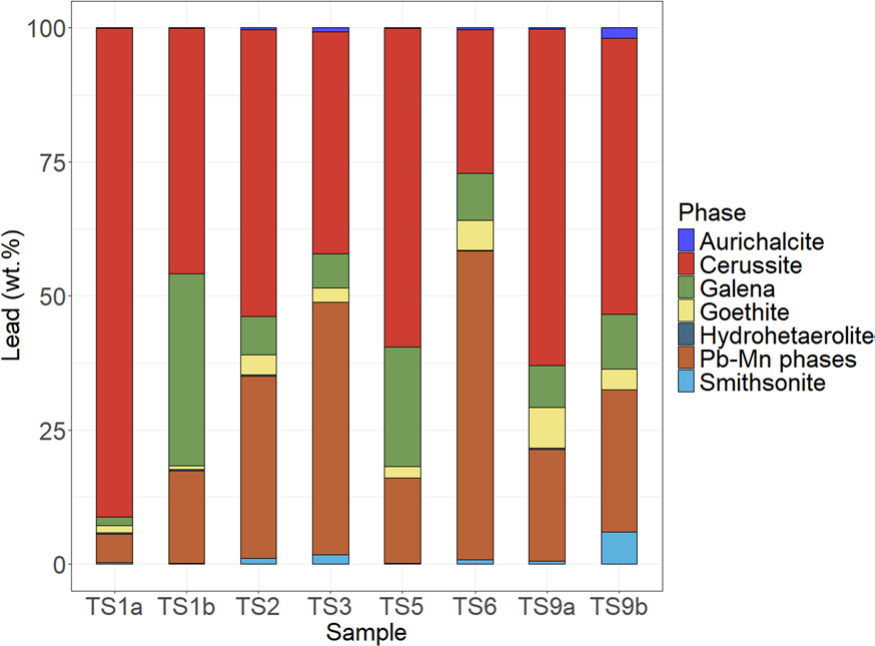
Lead deportment of Stirling tailings samples (N = 8)

The main sulfide minerals (chalcopyrite, galena, and tennantite) expected to host Cu or Pb within the deposit (Cleaver et al., 2021) were identified in Cu and Pb deportments (Figs. 1, 2). Chalcopyrite (CuFeS_2_) was the predominant Cu host in *all* samples, hosting between 48 and 86% of Cu. The second largest proportion of Cu was oxide-hosted (Pb–Mn phases, goethite, and hydrohetaerolite) in four samples (TS2, TS3, TS6, TS9a), and sulfosalt-hosted (tennantite) in three samples (TS1a, TS1b, TS5). The remaining sample, TS9b, had a substantial proportion of carbonate-hosted Cu, with 23 wt.% Cu of the sample hosted in aurichalcite and 4 wt.% in smithsonite, TS9b had the lowest proportion of sulfide-hosted Cu. Pyrite-poor samples, TS1b and TS5, had the highest proportion of sulfide-hosted Cu (chalcopyrite and tennantite).

**Fig. 3:**
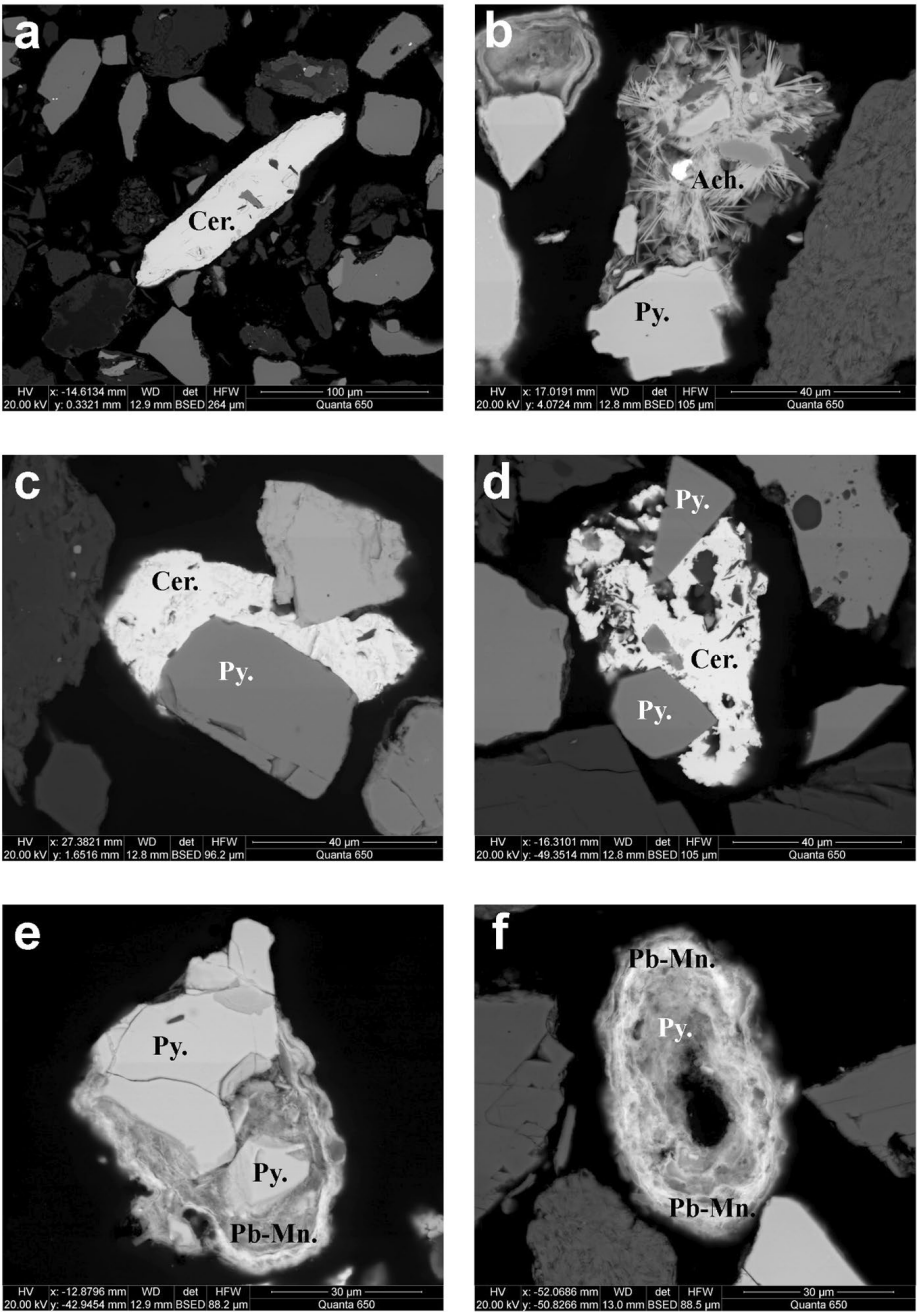
BSE (SEM) images of grains, within sieved (< 63 µm) Stirling tailings, hosting elements of interest. **a** elongate cerussite (Cer.) grain that will have passed the 63 µm sieve. **b** Needles of aurichalcite (Ach.). **c** and **d** pyrite (Py.) partially encapsulated by cerussite. **e** pyrite with a partial rim of Pb-Mn oxide phases (Pb-Mn.). **f** shows a weathered grain of pyrite, partially oxidised to Pb-Mn oxide phases

Lead deportment is shown in Fig. 2. Carbonates were the dominant Pb host in six of eight samples, specifically cerussite (PbCO_3_) which hosted between 26.8 and 91.2 wt.% Pb of a sample. Cerussite was chiefly seen, in BSE imaging, as liberated grains or as a cement often around pyrite (Fig. 3). The dominant lead host in the remaining two samples was Pb-Mn oxide phases, which typically occurred as a rim around pyrite (Fig. 3). The proportion of sulfide-hosted Pb ranges between 1.5 and 36 wt.% Pb of a sample: pyrite-poor samples had substantially more (22–36 wt.%) compared to pyrite-rich (1.5–10 wt.%). Aurichalcite hosts 0.1–1% of Pb in four samples, < 1% in three samples, and 2% in one sample, TS1b.

Copper hosts contrasted with Pb hosts, in that they were predominated by a more reduced phase, the sulfide chalcopyrite; Pb hosts were predominated by secondary minerals, cerussite and Pb-Mn phases, a carbonate and oxide(s), respectively. Pyrite-poor samples had the highest proportion of sulfide-hosts for both Cu (chalcopyrite and tennantite) and Pb (galena), though difference in proportion was minimal for copper.

### Particle-size distribution of copper and lead hosts

**Fig. 4:**
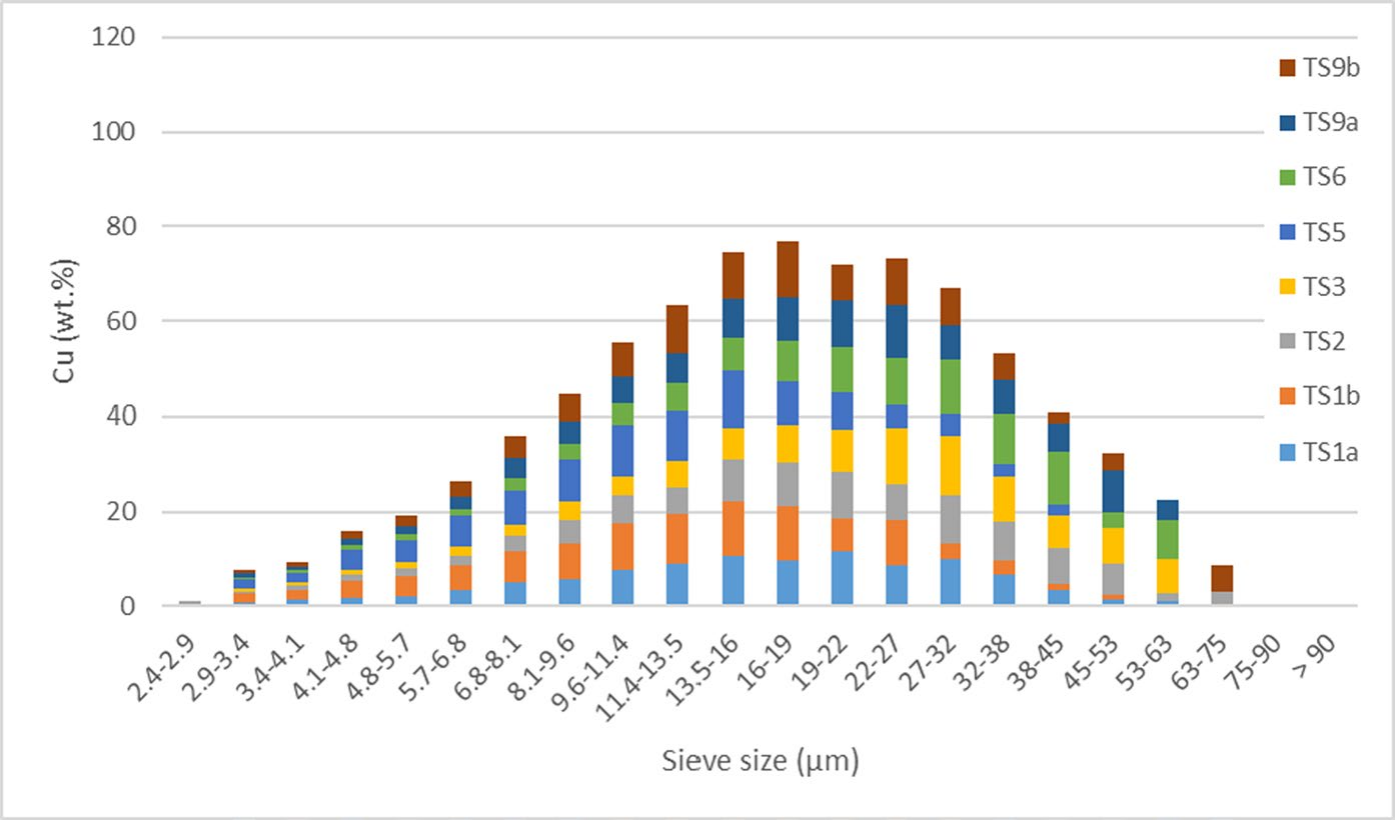
Particle-size distribution of copper hosts, by proportional of copper within a given sample (wt.%) against automated-mineralogy sieve size (µm)

**Fig. 5:**
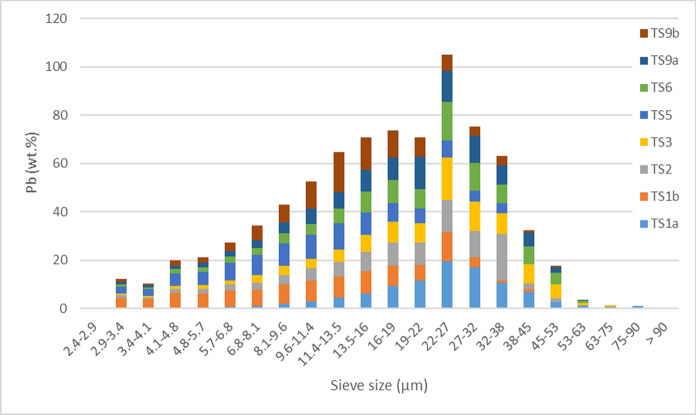
Particle-size distribution of lead hosts, by proportional of lead within a given sample (wt.%) against automated-mineralogy sieve size (µm)

Particle-size distribution across the sample set for Cu and Pb (wt.%) hosts is shown in Figs. 4 and 5, respectively. This was calculated from the automated mineralogy particle size distribution (wt.%) of Cu and Pb hosts multiplied by the proportion of the respective element (wt.%) the phase hosts in a given sample. Copper and lead are predominantly hosted in particles between 8.1 and 38 µm. A high concentration of Pb was hosted in the 22–27 µm sieve size, otherwise particle-size distribution between Cu and Pb is similar. Pyrite-poor samples, TS1b and TS5, were relatively fined grained, with over 50 wt.% of the Cu and Pb hosted in particles < 13.5 µm; comparably, in the remaining samples over 50 wt.% of Cu and Pb was hosted in particles < 22 µm.

Though samples were physically sieved with a 63-µm mesh (Cleaver et al., 2021), a small proportion of mineral grains, identified by SEM-MLA, were ≥ 63 µm (Table 4). Particles calculated as being ≥ 63 µm by SEM-MLA are thought to be resultant of elongated grains (Fig. 3, Fig. S5) that passed through the 63 µm sieve but have an area ≥ a 63-µm diameter circle (3117 µm^2^). Elongate grains of cerussite, chalcopyrite, Pb-Mn phases, smithsonite, sphalerite, tennantite, and unknown phases were identified. In 16 of 20 cases these grains are ≤ 2 wt.% of the phase for a given sample; in three cases 5–6 wt.%; and in one case, for sample TS9b, 51 wt.% of tennantite was present in one elongate grain ≥ 63 µm and < 75 µm.

### Gastric bioaccessibility

#### Stirling tailings

**Fig. 6:**
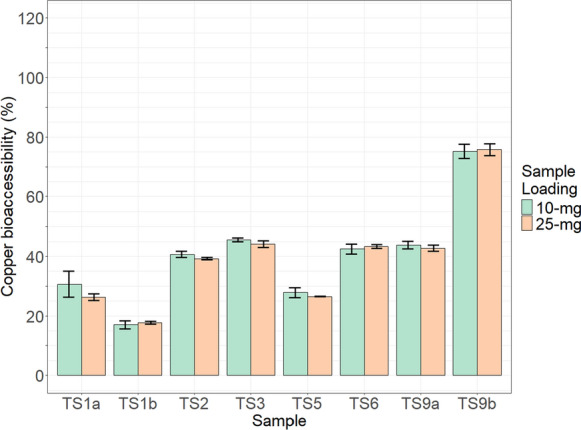
Copper bioaccessibility (%) for 10- and 25-mg sample loadings of Stirling tailings (N = 8). Error bars show standard deviation

**Fig. 7:**
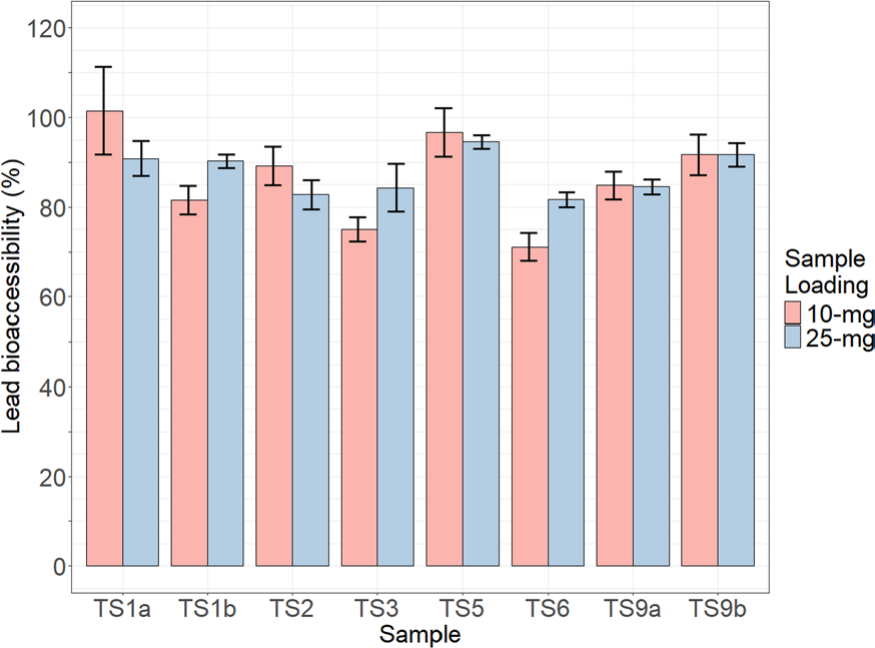
Lead bioaccessibility (%) for 10- and 25-mg sample loadings of Stirling tailings (N = 8). Error bars show standard deviation

Bioaccessibility results determined for Cu and Pb in the < 63-µm size fraction of tailings (*N* = 8) are presented in Table 6. Copper and lead bioaccessibility were not correlated with total concentration of the respective element within a sample. Spearman’s rank correlation was used to compute the relationship between total concentration of an element in a sample, and the bioaccessibility of that element: for Cu, ρ of − 0.07 and *p*-value of 0.882; for Pb, ρ of 0.05 and *p*-value of 0.935. For any given sample, Cu bioaccessibility was less than the respective sample’s Pb bioaccessibility (Figs. 6, 7). Copper and lead bioaccessibility had a negligible correlation between one another, with a Pearson correlation coefficient of − 0.12.

**Table 6:** Mean bioaccessibility results of triplicates for Stirling tailings (*N* = 8)

Sample ID	TS1a	TS1b	TS2	TS3	TS5	TS6	TS9a	TS9b
Copper % bioaccessible								
10-mg loading	31 ± 4.4	17 ± 1.3	41 ± 1.0	46 ± 0.7	28 ± 1.7	43 ± 1.7	44 ± 1.3	75 ± 2.4
25-mg loading	26 ± 1.1	18 ± 0.5	39 ± 0.4	44 ± 1.1	27 ± 0.1	43 ± 0.6	43 ± 1.0	76 ± 2.0
Lead % bioaccessible								
10-mg loading	101 ± 9.8	82 ± 3.2	89 ± 4.3	75 ± 2.7	97 ± 5.4	71 ± 3.1	85 ± 3.1	92 ± 4.6
25-mg loading	91 ± 3.9	90 ± 1.5	83 ± 3.3	84 ± 5.3	95 ± 1.5	82 ± 1.7	85 ± 1.7	92 ± 2.6

Copper bioaccessibility results for both sample loadings ranged from 17 to 76%; the range in Cu bioaccessibility when the 10-mg and 25-mg loadings for a sample were averaged was 18–76%. Solid-to-fluid ratios of extractions (0.2 and 0.5 g/L) had little impact, 0.6–4.4%, on Cu bioaccessibility, with seven of eight samples having had a difference < 1.5%. The percentage of Cu extracted in the 25-mg loading is comparable to that of the smaller 10-mg loading (Table 6), as Cu bioaccessibility values between sample loadings overlap for six of eight samples when standard deviation is accounted for; for loadings of samples TS2 and TS3 there is a 0.6% and 0.3% difference respectively between their 10- and 25-mg loadings when standard deviation is accounted for. Saturation of Cu in solution was either not present or had insubstantial impact. TS9b has the highest values for Cu bioaccessibility, 76% for the 25-mg loading; the next highest values seen in samples TS3, TS6, TS9a with bioaccessibility values between 43–46%. The two pyrite-poor samples (TS1b, TS5) have low Cu bioaccessibility. Mean Cu bioaccessibility, calculated from the two sample loadings, had a very strong positive correlation with the proportion of Cu hosted in carbonates and oxides (as opposed to sulfide and sulfosalt phases): Spearman’s rank correlation computed a ρ of 1, and *p*-value of < 0.001; or, put conversely, a strong negative correlation between Cu bioaccessibility and the proportion of Cu hosted in sulfide and sulfosalt phases.

Lead bioaccessibility results for both sample loadings ranged from 71–101%. The 10-mg sample loading for TS1a exceeded 100% bioaccessible, this may be consequent of heterogeneity between the subsample of TS1a used in elemental analysis and to determine bioaccessibility. The range when 10-mg and 25-mg loadings for a sample were averaged was 76–96% Pb bioaccessible, which is a substantially narrower range than seen for Cu in these samples. Saturation of Pb in the extraction solution in unlikely, as with standard deviation accounted for the percentage of Pb extracted between loadings of a given sample overlaps (seen in the error bars of Fig. 7) or has a higher percentage extracted in the 25-mg loading. Lead bioaccessibility (averaged from sample loadings) had a strong positive correlation with the proportion of Pb hosted within cerussite; Spearman’s rank correlation computing ρ of 0.74 and a *p*-value of 0.046. However, Pb bioaccessibility had a very weak positive correlation with carbonate- *and* oxide-hosted Pb; Spearman’s rank correlation returning a ρ of 0.02, and *p*-value of 0.977. Lowest and second lowest Pb bioaccessibility (76% and 80%) occurred in samples TS6 and TS3 respectively, which had respectively low proportions of cerussite-hosted Pb (27 wt.% Pb, and 41 wt.% Pb); highest Pb bioaccessibility from the average of both sample loadings (96%) occurred in sample TS1a, which had the highest proportion of cerussite-hosted Pb, 91 wt.% Pb. Pyrite-poor samples (TS1b, TS5) had comparable Pb bioaccessibility to other samples.

The average bioaccessibility of 10- and 25-mg sample loadings for both Cu and Pb did not show evidence of non-normality when tested with the Shapiro–Wilk normality test, with *p*-values of 0.27 and 0.60 for Cu and Pb respectively. In six samples, Pb bioaccessibility had higher variance between sample loadings than Cu; difference in bioaccessibility (%) between sample loadings ranged from 0.6–4.4 for Cu, and 0.3–10.6 for Pb.

## Discussion

### Elemental analysis

Difference in Cu and Pb concentrations between samples in our study and Cleaver et al. (2021) was likely consequent of sample (and sub-sample) heterogeneity, which could be expected for mine wastes (Surrette et al., 2024; Vriens et al., 2020). Elemental analysis in this study and Cleaver et al. (2021) both used aqua regia digest, though Cu and Pb concentrations were determined by ICP-OES in this study and ICP-MS in Cleaver et al. (2021). The mean copper concentration of the tailings is two orders of magnitude higher than the industrial CCME soil guideline for the protection of the environment and human health; the mean lead concentration for the tailings is one order of magnitude higher than the industrial CCME soil guideline (Canadian Council of Ministers for the Environment, 2007). Although the tailings are not expected to meet CCME guidelines, the guidelines provide a context for understanding risk given that the abandoned site is accessed by the public.

### Bioaccessibility and mineralogy

Difference between copper and lead gastric bioaccessibility, 42% and 86% median bioaccessibility respectively, was likely due to the extent of alteration of primary minerals hosting Cu and Pb, and thus the proportion of oxidation products (carbonates and oxides) that hosted Cu and Pb. On average, 15 wt.% Cu was hosted in carbonates and oxides, compared 87 wt.% for Pb; disparity between the proportion of oxidation products can be explained by the varying susceptibility of sulfide minerals to oxidation. Moncur et al. 2009 suggested that chalcopyrite is more resistant to oxidation than galena. Furthermore, carbonates and oxides are known to be highly bioaccessible phases and, conversely, sulfides are known to have low bioaccessibility (Drexler & Brattin, 2007; Driscoll et al., 2011; Helser et al., 2022). Copper bioaccessibility accorded with this general ranking of bioaccessible phases; higher Cu bioaccessibility occurred in samples with less sulfide-hosted copper (thus more carbonate- and oxide-hosted copper). Highest Cu bioaccessibility occurred in sample TS9b, which was collected on the lee side of a small dune at a 10–25 cm depth (Cleaver, 2020); this sample, despite being taken at depth, has the highest proportion of copper hosted in oxidation products, with over double the proportion of Cu hosted in oxides and carbonates than sample TS9a, which was collected directly above it. Sample TS9b may have been surficial until buried by dust accumulation.

**Fig. 8:**
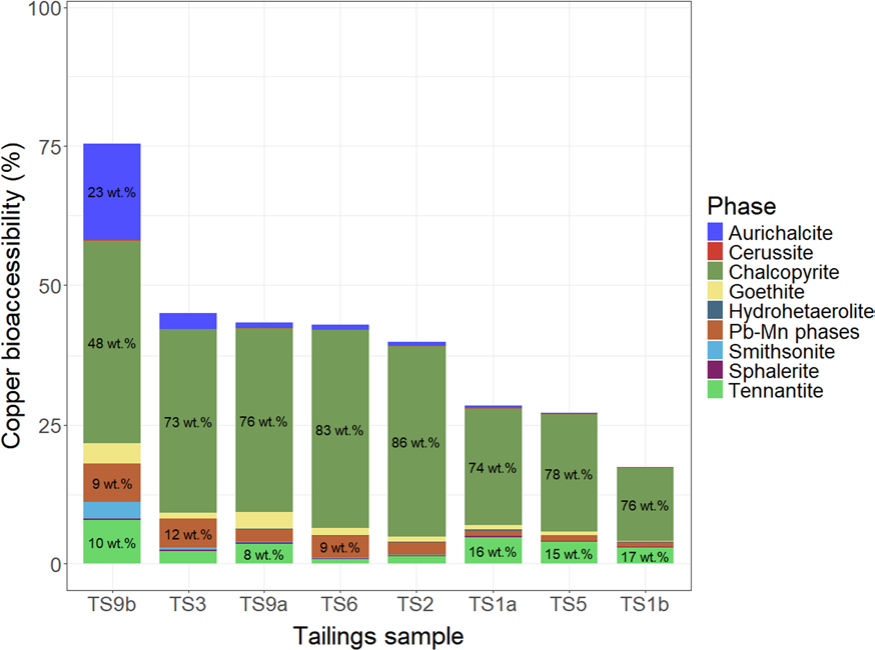
Copper deportment (wt.%) normalised to the copper bioaccessibility (%) of the sample. Labels for Cu deportment included where phase hosts > 7 wt.% of Cu in the sample

**Fig. 9:**
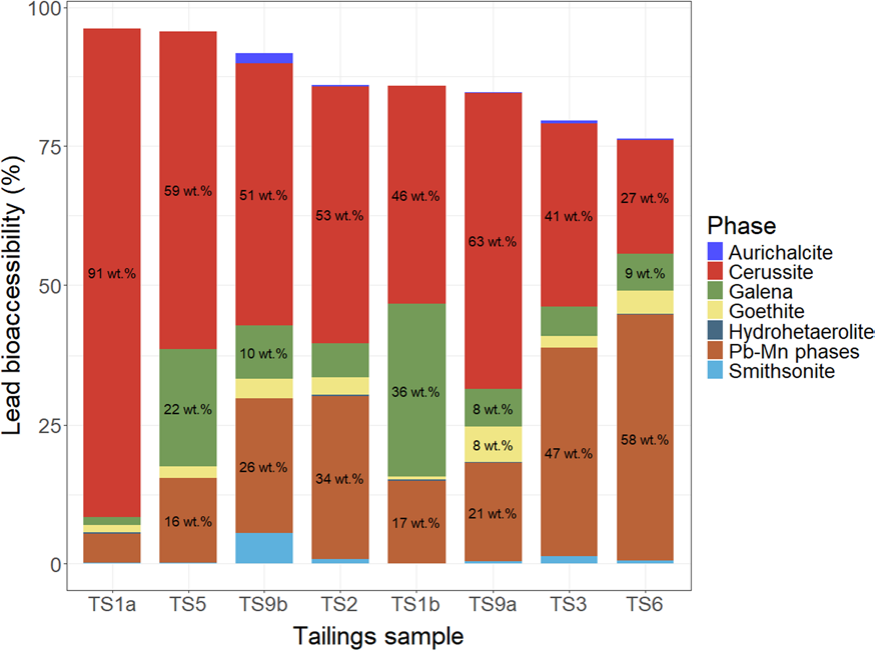
Lead deportment (wt.%) normalised to the lead bioaccessibility (%) of the sample. Labels for Pb deportment included where phase hosts > 7 wt.% of Pb in the sample

However, Pb bioaccessibility did not correlate with the proportion of Pb hosted in oxidation products but instead, specifically, the proportion of cerussite, which was the only mineral positively correlated with lead bioaccessibility. The highest Pb bioaccessibility, from the average of both sample loadings (96%), occurred in TS1a, the sample with the highest proportion of cerussite-hosted Pb (91 wt.%). However, factors others than speciation appear to have impacted bioaccessibility, as three samples, TS1b, TS2, and TS9a, had Pb bioaccessibility values that ranged between 85–86% bioaccessible (from an average of the sample loadings), yet the proportion of cerussite-hosted Pb ranged from 46 to 63 wt.%. Particle-size distribution of cerussite was similar in these three samples (Fig. S6). Lack of correlation between Pb bioaccessibility and Pb concentrations accords to previous findings (Yang et al., 2003) wherein variations in concentration of Pb were not a significant factor on Pb bioaccessibility from testing that used pH 2, and solid-to-fluid ratios of 10 and 25 g/L (Figs. 8, 9).

The broader range of Cu bioaccessibility (17–76%) compared to Pb (76–96%) may be partially attributable to the difference between the minimum and maximum wt.% of the respective element hosted in phases that strongly correlated with bioaccessibility. For Pb, the sample with the most carbonate-hosted lead (92 wt.% Pb) had a proportion ~ 3.3 times greater than the sample with the least (28 wt.% Pb); for Cu, the sample with the most amount of carbonate- and oxide-hosted copper (41 wt.% Cu) had a proportion ~ 6.8 times greater than the least (6 wt.% Cu).

Compared to un-sieved tailings, the extent of sulfide oxidation, and thus bioaccessibility, may be higher in the sieved (< 63 µm) samples, as this smaller size-fraction is expected to have been more susceptible to sulfide oxidation (Blowes et al., 2014). In soils, a general trend of higher bioaccessibility for potentially toxic elements with decreasing particle size has been noted (Li et al., 2021).

The greater range seen in initial pH values, prior to pH adjustment, between samples compared to the narrow range seen in final pH values (Table 1) may be indicative of sample heterogeneity. The reduction in pH variation was to help isolate mineralogy as a factor on bioaccessibility. The pH maintained at 1.5 is likely to have provided a moderately conservative result for bioaccessibility of the Stirling tailings as, in general, bioaccessibility simulations using extraction solutions of lower pH return higher bioaccessibility values (Boros et al., 2017; Hedberg et al., 2010; Koch et al., 2013). Sensitivity to pH for gastric bioaccessibility was noted in Boros et al. (2017), who attribute their relatively low gastric bioaccessibility for Pb (65%) in NIST 2710 as consequent of a relatively high pH (1.8) gastric extraction solution, compared to studies with lower pH (1.4–1.5) gastric extraction solutions with Pb bioaccessibility results for NIST 2710 in the mid-70% range (Dodd et al., 2013; Drexler & Brattin, 2007; Ellickson et al., 2001; Rasmussen et al., 2011).

Following from the results of this study, there is potential for future work that isolates factors impacting bioaccessibility. This includes investigation of particle size and surface area on the rate and extent of metal release; the impact of less common secondary minerals (e.g. aurichalcite) on metal release, for which fundamental solubility information, such as solubility constants, if determined, may indicate human-health risk; and further bioaccessibility testing on pure, single-phase copper minerals such as in Driscoll et al. (2011).

## Conclusions

This study investigated Cu and Pb bioaccessibility, and Cu-host and Pb-host mineralogy in tailings samples previously collected by (Cleaver et al., 2021) from an uncovered, subaerial tailings impoundment at the abandoned Stirling Mine, Nova Scotia, Canada. As in the study that collected the samples (Cleaver et al., 2021), both Cu and Pb concentrations in Stirling tailings substantially exceeded respective CCME Parkland guidelines, and CCME Industrial guidelines. Copper was predominately hosted in chalcopyrite. Copper bioaccessibility increased with increasing proportions of copper hosted in oxidation products, notably aurichalcite. Lead was predominately hosted in cerussite in six samples, as well as an unidentified Pb-Mn phase, thought to be PbMn_3_O_6_(OH)_2_, in the two remaining samples. Lead bioaccessibility had negligible correlation to proportions of lead hosted in oxidation products relative to primary galena, but a strong positive correlation to the presence of one oxidation product, cerussite (PbCO_3_). The study supports the importance of mineralogy as a factor on bioaccessibility, and highlights that, in these mine wastes, the predominant mineral family for elements of concern differs. Use of an uncovered, subaerial impoundment for sulfide tailings likely exacerbates the human-health risk of the tailings if ingested due to formation of highly bioaccessible oxidation products.

## Supplementary Information

Below is the link to the electronic supplementary material.

Supplementary file1 (DOCX 1284 KB)

## Data Availability

Data is provided within the manuscript or supplementary information files.
