# Supplementary material for: Increased human health risks at a legacy mine site: copper and lead bioaccessibility of oxidised tailings
Source: Environ Geochem Health. 2025 Jul 21;47(8):326. doi: 10.1007/s10653-025-02498-6 (PMC12279896; doi:10.1007/s10653-025-02498-6)
Supplement: Supplementary file 1 — Supplementary file1 (DOCX 1284 KB) [file 10653_2025_2498_MOESM1_ESM.docx]

**Supplementary Information**

**Increased human health risks at a legacy mine site: copper and lead bioaccessibility of oxidized tailings**

Sean McHale^a*^

Heather E. Jamieson^a^

Amy E. Cleaver^b^

Philippa Huntsman^b^

^a^*Queen’s University, Department of Geological Sciences and Geological Engineering, Kingston, K7L 3N6, Canada*

*^b^CanmetMINING, Natural Resources Canada, Ottawa, K1A 0G1, Canada*

*Corresponding author

E-mail address: 19stm3@queensu.ca


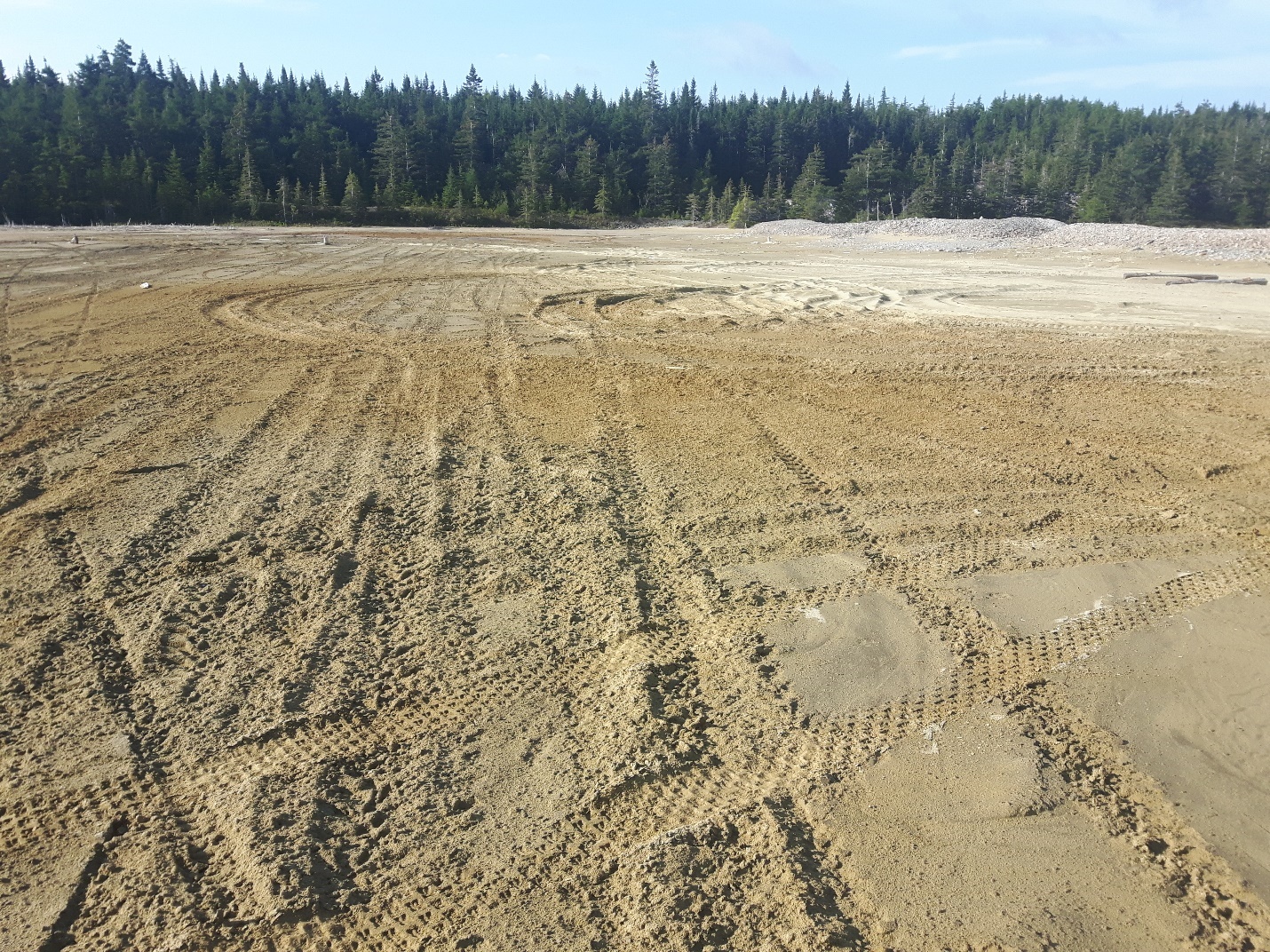


**Fig. 1** Stirling tailings impoundment pictured with all-terrain vehicle tracks present. Photo credit Amy Cleaver


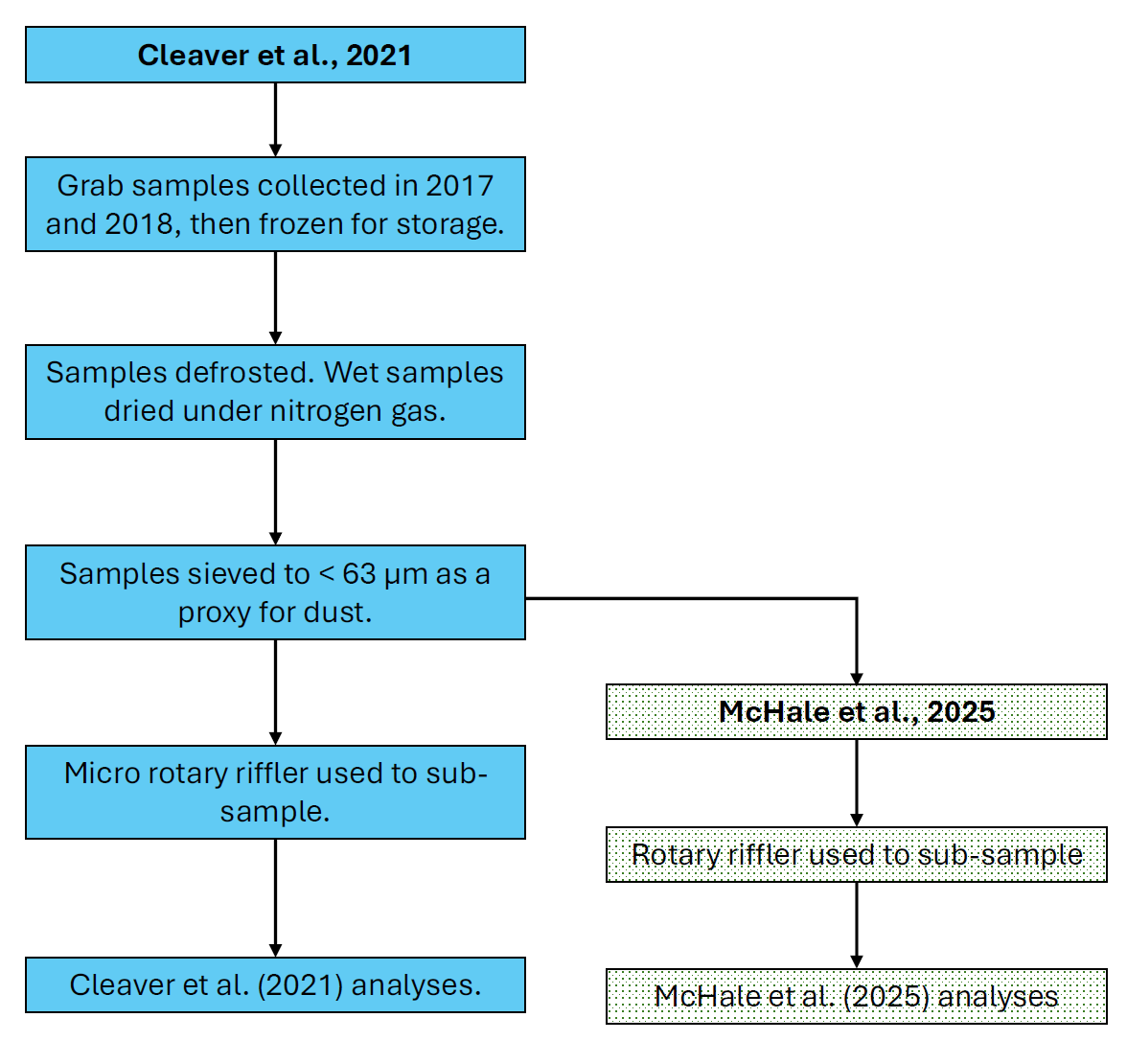


**Fig. 2** Flow chart summarising sample preparation

**Table 1** Certified reference material types

| CRM | Type |
| --- | --- |
| 900-EMR-512 | Solution |
| IV-71A | Solution |
| SOIL-A | Solution |
| SOIL-B | Solution |
| TM-25.6 | Solution |
| TMDA-51.5 | Solution |
| TMDA-54.6 | Solution |
| TMDW | Solution |
| WAVECAL | Solution |

**Table 2** Percent extracted of certified values in certified reference materials used during elemental analysis of tailings

| CRM | Type | % recovered for CRMs in tailings elemental analysis | | | | | | |
| --- | --- | --- | --- | --- | --- | --- | --- | --- |
|  |  | As | Cd | **Cu** | Ni | **Pb** | Sb | Zn |
| WAVECAL | Solution | 104 | - | - | - | - | - | - |
| SOIL-B | Soil | 99 | - | 97 | - | 95 | - | 100 |
| SOIL-A | Soil | - | - | - | - | 96 | - | 98 |
| TMDA-54.6 | Solution | - | 111 | 102 | 105 | 105 | - | 105 |
| TMDW | Solution | - | 113 | - | 102 | - | 110 | - |
| TMDW | Solution | - | 105 | - | 102 | - | 99 | - |
| TMDA-54.6 | Solution | - | 103 | - | 102 | - | - | - |
| TM-25.6 | Solution | - | 111 | - | 108 | - | 109 | - |
| TM-25.6 | Solution | - | 106 | - | 107 | - | 100 | - |

**Table 3** Relative percent difference for copper and lead in analytical duplicate for tailings elemental analysis

| Tailings duplicate | Cu RPD | Pb RPD |
| --- | --- | --- |
| TS1a | 3.90% | 3.54% |

**Table 4** Percent extracted for given elements in certified reference materials in bioaccessibility extract-solution analysis for NIST 2710a

| CRM | % recovered for CRMs analyzed alongside NIST 2710a extract solutions | | | | |
| --- | --- | --- | --- | --- | --- |
|  | Cd | Cu | Ni | Pb | Zn |
| SOIL-B |  | 98 |  | 92 | 98 |
| IV-71A | 102 | 103 |  | 102 | 102 |
| 900-EMR512 | 102 |  |  | 102 | 103 |
| SOIL-A |  | 101 |  |  | 92 |
| TMDW | 105 | 115 | 107 | 95 | 108 |
| TMDA-51.5 | 104 | 101 |  |  |  |
| TMDW | 102 | 100 | 99 | 93 | 104 |
| TMDA-51.5 | 106 | **111** |  |  |  |

**Table 5** NIST 2710a bioaccessibility results with comparison to other studies and method summaries

| **Data from** | Bioaccessibility (%) | | | | | # of stages | Loading (mg) | Gastric volume (mL) | °C | Incubation (h) | Agitation (rpm) | Extraction solution | Gastric pH |
| --- | --- | --- | --- | --- | --- | --- | --- | --- | --- | --- | --- | --- | --- |
|  | **Cd** | **Cu** | **Ni** | **Pb** | **Zn** |  |  |  |  |  |  |  |  |
| 10 mg this study | 62 ± 4 | 82 ± 7 | 250 ± 19 | 87 ± 12 | 48 ± 4 | 1 | 10 | 50 | 37 | 2 | 100 | 0.07M HCl | 1.5 |
| 25 mg this study | 55 ± 0.7 | 78 ± 1 | 120 ± 7 | 76 ± 2 | 46 ± 1 | 1 | 25 | 50 | 37 | 2 | 100 | 0.07M HCl | 1.5 |
| Boros et al. (2017) Gastric | 46 | 53 | 13 | 46 | 42 | 1 | 1000 | 100 | 37 | 1 | 28 | Pepsin, sodium citrate, malic acid disodium salt, lactic acid (85%), acetic acid, HCl | 1.8 |
| Boros et al. (2017)  GI | 23 | 37 | 8 | 5 | 12 | 2 | 1000 | 100 | 37 | 5 | 28 | Above + bile salts and pancreatin | 1.8 |
| Xia et al. (2016)  Gastric | 45 | - | - | - | - | 1 | 300 | 30 | 37 | 1 | 30 | Too long to list. As in Wragg et al. (2009) | 1.2 |
| Xia et al. (2016) GI | 26 | - | - | - | - | 3 | 300 | 30 | 37 | 5 | 30 | As in Wragg et al. (2009) | 1.2 |
| Nelson et al. (2013) water incubation | - | - | - | 64 | - | 1 | 1000 | 100 | 37 | 1 | 30 | 0.4M glycine + HCl | 1.5 |
| Nelson et al. (2013)  air incubation | - | - | - | 65 | - | 1 | 1000 | 100 | 37 | 1 | 30 | 0.4M glycine + HCl | 1.5 |
| Paltseva et al. (2018) | - | - | - | 65 | - | 1 | 1000 | 100 | 37 | 1 | 30 | 0.4M glycine + HCl | 1.5 |
| Ono et al. (2016) Gastric | 33 | - | - | 33 | - | 1 | 1000 | 100 | 37 | 1 | 100 | Ultrapure water, pepsin, citrate, malate, lactic acid, acetic acid, HCl | 2.5 |
| Ono et al. (2016) Intestinal | 33 | - | - | 1 | - | 2 | - | - | 37 | 3 | 100 | Ultrapure water, pepsin, citrate, malate, lactic acid, acetic acid, HCl | 2.5 |
| Pelfrêne and Douay (2018) Gastric | 93-108 | - | - | 88–109 | - | 1 | 600 | 22.5 | 37 | 1 | "end over end" | As in Wragg et al. (2009) | 1.2 |
| Pelfrêne and Douay (2018) GI | 89-109 | - | - | 88–109 | - | 3 | 600 | 22.5 | 37 | 5 | "end over end" | As in Wragg et al. (2009) | 1.2 |
| Coa et al. (2019) | - | - | - | 88.4 | - | Pb value (88.4) is an average from three methods. Separate values not given. | | | | | | | |


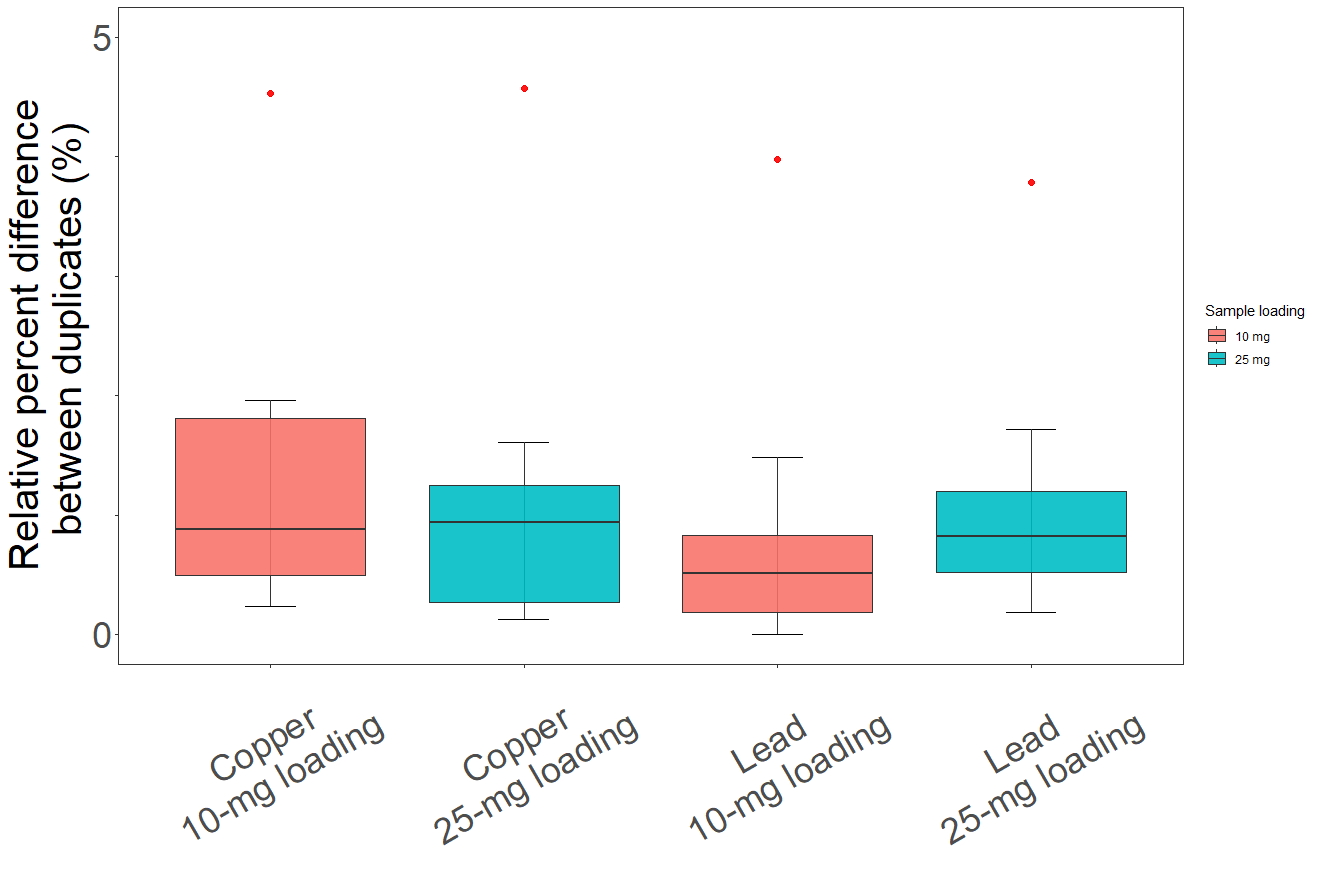


**Fig. 3** Box and whisker plot of relative percent difference for bioaccessibility-extract solution duplicates

**Table 6** Percent recovered for given elements in certified reference materials, analysed alongside bioaccessibility-solution aliquots for sample TS9b

| CRM | % recovered for CRMs analysed alongside TS9b extract solutions | | | | | |
| --- | --- | --- | --- | --- | --- | --- |
|  | As | Cd | Cu | Pb | Sb | Zn |
| SOIL-B |  |  | 99 | 96 |  | 98 |
| IV-71A |  | 99 |  | 100 |  | 100 |
| 900-EMR512 |  | 101 |  | 101 | 100 | 102 |
| TMDW | 102 | 93 | **77** | 90 | 99 | 114 |
| TMDW | 99 | 94 | 83 | 92 | 108 | 118 |
| TMDA-51.5 | 102 | 95 | 93 |  | 99 |  |
| TMDA-51.5 | 99 | 95 | 95 |  | 99 |  |


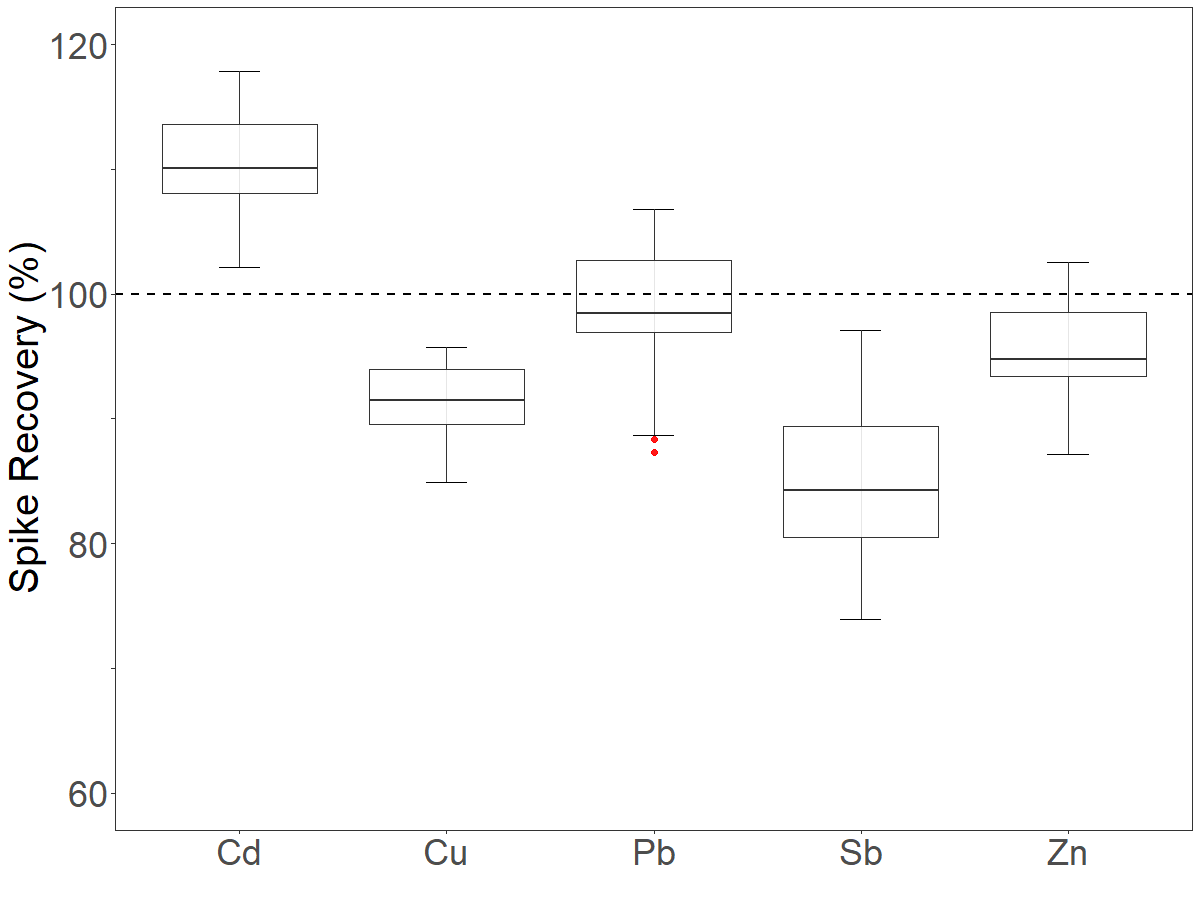


**Fig. 4** Box and whisker plot for percentage of copper, and lead recovered from positive controls in bioaccessibility testing

**Table 7** Average blank values (ppb) for bioaccessibility analyses. Nine blanks were run per sample per element

|  | Sample | | | | | | | |
| --- | --- | --- | --- | --- | --- | --- | --- | --- |
|  | TS1a | TS1b | TS2 | TS3 | TS5 | TS6 | TS9a | TS9b |
| Cu (ppb) blank average (n = 9) | 2.898 | 0.419 | 2.236 | 1.039 | 0.314 | 0.045 | 10.434 | 0.170 |
| Pb (ppb) blank average (n = 9) | 12.622 | 1.678 | 1.59 | 9.624 | 1.408 | 2.733 | 25.687 | 3.638 |

Table 8 Summary of elemental analysis for Stirling tailings, with guideline values from the Canadian Council for Ministers of the Environment (CCME)

| Element | Avg. this study*  Sieved < 63µm (ppm) | Avg. Cleaver et al. 2021* Sieved < 63 µm (ppm) | CCME Soil Guideline Parkland (ppm) | CCME Soil Guideline Industrial  (ppm) |
| --- | --- | --- | --- | --- |
| Number of samples | *N* = 8 | *N* = 7 |  |  |
| Zn | 15,611 ± 7,419 | 13,529 ± 4,626 | 250 | 410 |
| **Pb** | **5,226 ± 2470** | 3,881 ± 1,102 | 140 | 600 |
| **Cu** | **1,549 ± 328** | 1,631 ± 277 | 63 | 91 |
| As | 222 ± 43 | 232 ± 59 | 12 | 12 |
| Sb | 79 ± 15 | 89 ± 12 | 20 | 40 |
| Cd | 39 ± 18 | 38 ± 10 | 10 | 22 |

Modal mineralogy

All tailings samples were dominated by sulfide phases. Sample TS1b and TS5, classified as pyrite-poor, were constituted of 62 and 64 wt.% sulfide phases; the five pyrite-rich tailings ranged between 79–83 wt.% sulfides. The remaining sample, TS9b, was 71 wt.% sulfide phases. The primary sulfide was pyrite (FeS_2_), samples ranged from 57–81 wt.% pyrite. Dolomite (CaMg(CO_3_)_2_) and quartz (SiO_2_) are present in all samples, ranging from 6.5–9.5 wt.% and 2.5–5.5 wt.% respectively. Carbonate minerals, including Pb and Cu carbonates ranged from 8–14 wt.% of the sample: pyrite-rich tailings were 8–9wt. % carbonate phases, pyrite-poor were 11–12 wt.%, and TS9b 14 wt.%.

**Table 9** Modal mineralogy comparison between this study and Cleaver (2021). Standard deviation represents sample heterogeneity with classification of pyrite-poor and pyrite-rich tailings

| Mineral | This study | | Cleaver et al. 2021 | | % change | |
| --- | --- | --- | --- | --- | --- | --- |
|  | Wt.% in Pyrite-rich Tailings | Wt. % in Pyrite-poor Tailings | Wt.% in Pyrite-rich Tailings | Wt.% in Pyrite-poor Tailings | Pyrite rich | Pyrite poor |
| Pyrite | 78 ± 2.8 | 58 ± 0.9 | 51 ± 6 | 22 ± 3 | 53 | 164 |
| Dolomite | 6.3 ± 0.2 | 9.6 ± 0.3 | 21 ± 4 | 29 ± 1 | -70 | -67 |
| Quartz | 2.7 ± 0.3 | 5.7 ± 0.6 | 9.5 ± 2 | 17 ± 2 | -72 | -66 |
| Talc | 1.5 ± 0.4 | 4.6 ± 0.05 | 3.2 ± 1 | 9.6 ± 2 | -53 | -52 |
| Magnesite | 1.1 ± 0.2 | 0.8 ± 0.2 | 1.7 ± 1 | 2.1 ± 0.1 | -35 | -62 |
| Sphalerite | 2.5 ± 1.5 | 3.8 ± 1.7 | 1.7 ± 1 | 1.8 ± 1 | 47 | 111 |
| Clay Minerals | 0.06 ± 0.01 | 0.2 ± 0.005 | 1.4 ± 0.7 | 1.4 ± 0.4 | -96 | -86 |
| Barite | 2.2 ± 0.2 | 2.5 ± 0.2 | 1.4 ± 0.2 | 1.1 ± 0.4 | 57 | 127 |
| Amphiboles | 0.5 ± 0.01 | 3.5 ± 0.01 | 1.3 ± 0.3 | 3.2 ± 0.5 | -62 | 9 |
| Clinochlore | 0.3 ± 0.06 | 0.6 ± 0.02 | 1.2 ± 0.2 | 2.2 ± 0.1 | -75 | -73 |
| Chlorite | 0.5 ± 0.1 | 0.8 ± 0.03 | 1.0 ± 0.2 | 2.0 ± 0.2 | -50 | -60 |


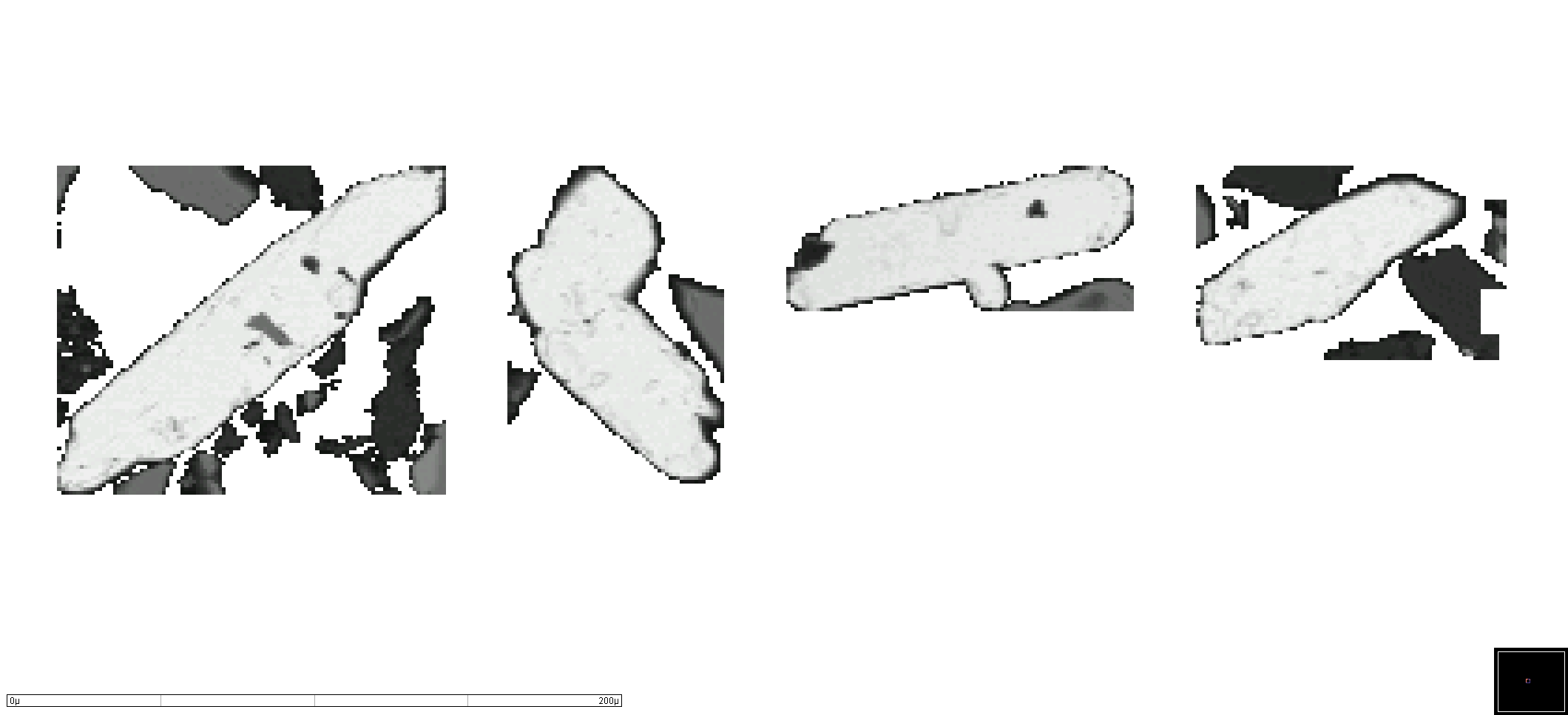


**Fig. 5** Sample TS1a, largest elongate cerussite grains. SEM-MLA scale bar = 200 µm


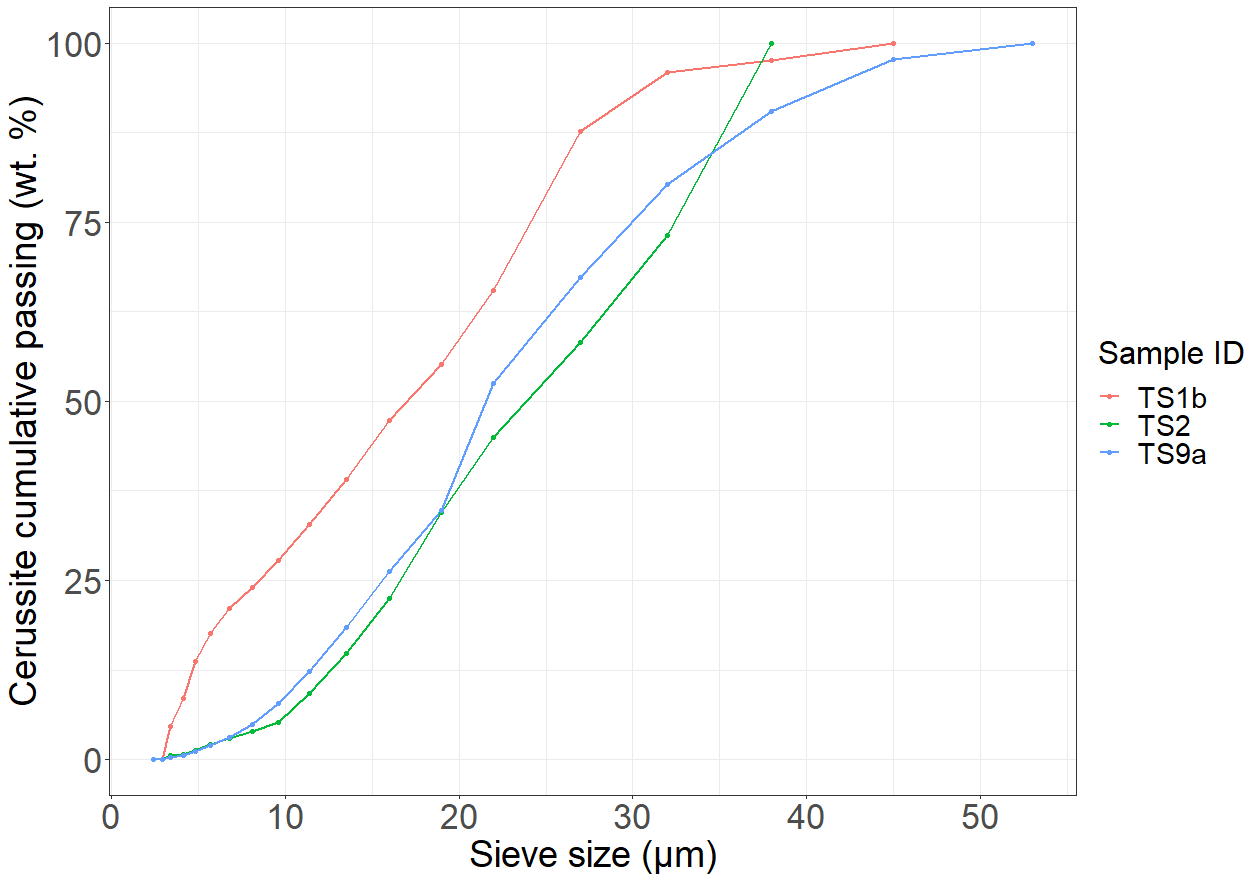


**Fig. 6** Particle size of cerussite, determined by automated-mineralogy software, in samples TS1b, TS2, and TS9a

Reference list

Boros, K., Fortin, D., Jayawardene, I., Chénier, M., Levesque, C., & Rasmussen, P. (2017). Comparison of Gastric versus Gastrointestinal PBET Extractions for Estimating Oral Bioaccessibility of Metals in House Dust. *International Journal of Environmental Research and Public Health, 14*(1), 92. doi:10.3390/ijerph14010092

Cao, P., Fujimori, T., Juhasz, A., & Takaoka, M. (2019). Bioaccessibility of Arsenic and Lead in Polluted Soils Using Three In-vitro Gastrointestinal Simulation Models. *IOP Conference Series: Earth and Environmental Science, 265*, 012012. doi:10.1088/1755-1315/265/1/012012

Nelson, C. M., Gilmore, T. M., Harrington, J. M., Scheckel, K. G., Miller, B. W., & Bradham, K. D. (2013). Evaluation of a low-cost commercially available extraction device for assessing lead bioaccessibility in contaminated soils. *Environmental Science: Processes & Impacts, 15*(3), 573. doi:10.1039/c2em30789h

Ono, F. B., Penido, E. S., Tappero, R., Sparks, D., & Guilherme, L. R. G. (2016). Bioaccessibility of Cd and Pb in tailings from a zinc smelting in Brazil: implications for human health. *Environmental Geochemistry and Health, 38*(5), 1083-1096. doi:10.1007/s10653-015-9774-0

Paltseva, A., Cheng, Z., Deeb, M., Groffman, P. M., & Maddaloni, M. (2018). Variability of Bioaccessible Lead in Urban Garden Soils. *Soil Science, 183*(4), 123-131. doi:10.1097/ss.0000000000000232

Pelfrêne, A., & Douay, F. (2018). Assessment of oral and lung bioaccessibility of Cd and Pb from smelter-impacted dust. *Environmental Science and Pollution Research, 25*(4), 3718-3730. doi:10.1007/s11356-017-0760-1

Wragg, J., Cave, M., Taylor, H., Basta, N., Brandon, E., Casteel, S., . . . Van de Wiele, T. (2009). *Inter-laboratory trial of a unified bioaccessibility testing procedure*.

Xia, Q., Peng, C., Lamb, D., Mallavarapu, M., Naidu, R., & Ng, J. C. (2016). Bioaccessibility of arsenic and cadmium assessed for in vitro bioaccessibility in spiked soils and their interaction during the Unified BARGE Method (UBM) extraction. *Chemosphere, 147*, 444-450. doi:<https://doi.org/10.1016/j.chemosphere.2015.12.091>
